# In situ construction of flower-like nanostructured calcium silicate bioceramics for enhancing bone regeneration mediated via FAK/p38 signaling pathway

**DOI:** 10.1186/s12951-022-01361-5

**Published:** 2022-03-27

**Authors:** Peng Mei, Shengjie Jiang, Lixia Mao, Yijia Zhou, Kaijun Gu, Chen Zhang, Xudong Wang, Kaili Lin, Cancan Zhao, Min Zhu

**Affiliations:** 1 Department of Oral & Cranio-Maxillofacial Surgery, Shanghai Ninth People’s Hospital, College of Stomatology, Shanghai Jiao Tong University School of Medicine, National Clinical Research Center for Oral Diseases, Shanghai Key Laboratory of Stomatology & Shanghai Research Institute of Stomatology, Shanghai, China; 2 Department of General Dentistry, Shanghai Ninth People’s Hospital, College of Stomatology, Shanghai Jiao Tong University School of Medicine, National Clinical Research Center for Oral Diseases, Shanghai Key Laboratory of Stomatology & Shanghai Research Institute of Stomatology, Shanghai, China; 3 Shanxi Medical University School and Hospital of Stomatology, Taiyuan, China

**Keywords:** Flower-like nanostructure, Calcium silicate, Hydrothermal treatment, Bone regeneration, FAK/p38 signaling pathway

## Abstract

**Background:**

The repair of tissue defects has attracted considerable attention and remained a substantial challenge. Calcium silicate (CaSiO_3_, CS) bioceramics have attracted the interest of researchers due to their excellent biodegradability. Recent studies have demonstrated that nanoscale-modified bioactive materials with favorable biodegradability could promote bone tissue regeneration, providing an alternative approach for the repair of bone defects. However, the direct construction of biodegradable nanostructures in situ on CS bioceramics was still difficult.

**Results:**

In this study, flower-like nanostructures were flexibly prepared in situ on biodegradable CS bioceramics via hydrothermal treatment. The flower-like nanostructure surfaces exhibited better hydrophilicity and more significantly stimulated cell adhesion, alkaline phosphatase (ALP) activity, and osteogenic differentiation. Furthermore, the CS bioceramics with flower-like nanostructures effectively promoted bone regeneration and were gradually replaced with newly formed bone due to the favorable biodegradability of these CS bioceramics. Importantly, we revealed an osteogenesis-related mechanism by which the FAK/p38 signaling pathway could be involved in the regulation of bone mesenchymal stem cell (BMSC) osteogenesis by the flower-like nanostructure surfaces.

**Conclusions:**

Flower-like nanostructure surfaces on CS bioceramics exerted a strong effect on promoting bone repair and regeneration, suggesting their excellent potential as bone implant candidates for improving bone regeneration.

**Graphical Abstract:**

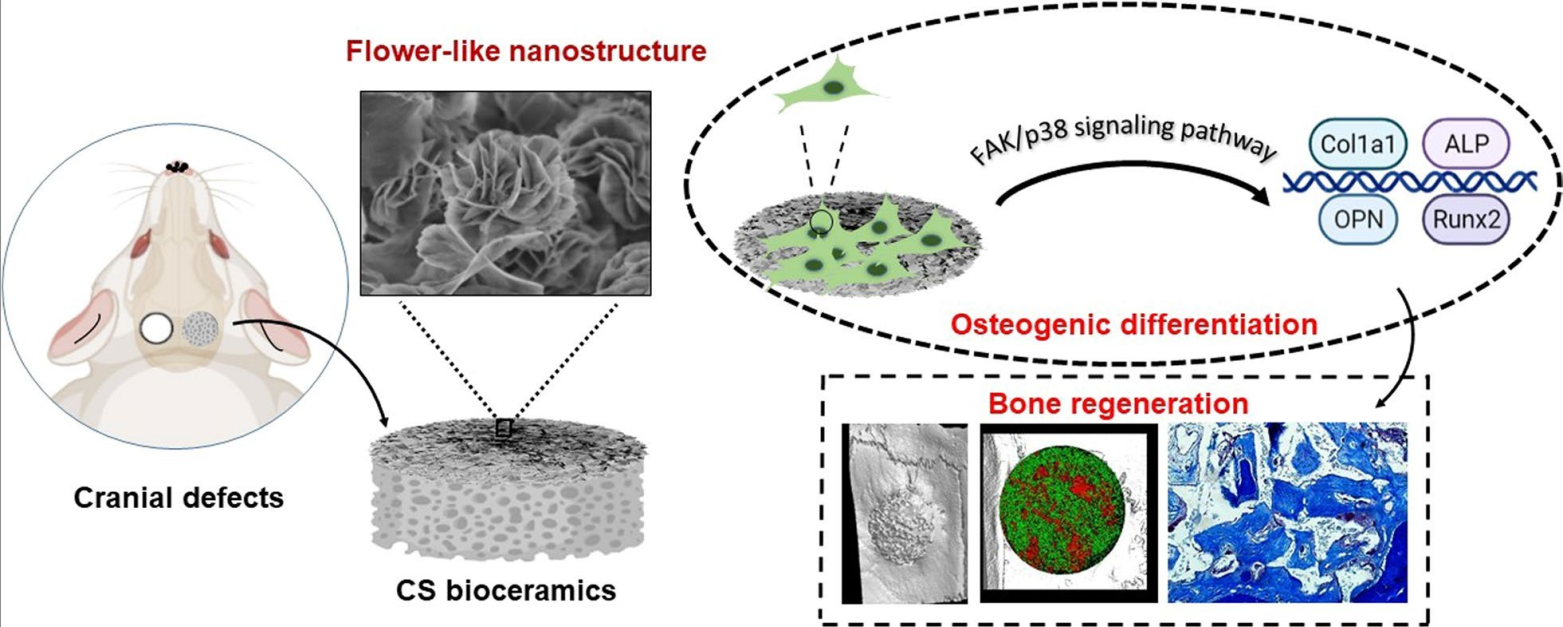

**Supplementary Information:**

The online version contains supplementary material available at 10.1186/s12951-022-01361-5.

## Introduction

Bioceramics have been widely applied in the field of bone regeneration and have been proven to have strong osteogenic properties. Nanoscale structures could alter the interaction of cells and bioactive materials and further influence cell behavior and cell fate, lead to the revolutionary application of these structures in biosensing, medical imaging, tissue regeneration and drug delivery [1–3]. Thus, many researchers have recently focused on designing and fabricating nanostructured surfaces and observing their effects on cellular behaviors [4–6]. Popat et al. found that nanostructured alumina ceramics prepared by a two-step anodization process exhibited favorable osteogenic properties compared with alumina ceramics without nanostructures [7]. In addition, nanorod structures were constructed successfully on the surface of hydroxyapatite (HA) bioceramics via hydrothermal treatment, and these nanorod structures significantly enhanced osteogenesis [8]. In general, nanostructures were constructed on bone repair materials with extremely low degradation rates, such as HA bioceramics, to enhance osteogenesis. It was well known that the appropriate biomaterials for bone regeneration should be resorbable and eventually be replaced with newly regenerated bone. Unfortunately, the degradability of bioceramic materials depended on their chemical composition and structures, which could not be changed by surface modification [9]. Because of the extremely low rates of bone repair material degradation, osteoblasts could not migrate into defects and may cause secondary damage during implant removal, leading to limitations in the clinical application of these materials [10].

Biodegradable bioceramics, especially calcium silicate (CS) bioceramics, have attracted considerable attention, particularly because of their excellent degradability compared with that of β-tricalcium phosphate (β-TCP) [9]. CS has a high solubility product constant (Ksp=2.5×10^−8^), and many studies have suggested that CS bioceramics could promote bone regeneration, mainly because of the release of bioactive Si ions during the biodegradation process [11, 12]; these Si ions could activate the bone morphogenetic protein 2 (Bmp2) signaling pathway, which was related to bone mineralization and regeneration [13–15]. During the biodegradation process, osteoblasts were recruited near the bioceramics and participated in the mineralization of the extracellular matrix (ECM). Furthermore, bioceramics promoted bone reconstruction with vessel ingrowth, and newly regenerated bone could replace bone repair materials during bioceramics degradation [10]. It was meaningful to construct nanostructures on biodegradable CS bioceramics. It was well established that blood vessels played a vital role in the processes of bone tissue repair and regeneration, and some studies have investigated the effect of CS bioceramics on angiogenesis [16–18]. Li H et al. found that CS bioceramics could stimulate the vascularization and angiogenesis of human umbilical vein endothelial cells (HUVECs), further showing the promising potential of CS bioceramics in bone repair and regeneration [19]. Therefore, CS bioceramics could be considered a promising candidate for bone repair and bone substitute materials.

To date, some methods have been reported for synthesizing nanoscale CS materials, such as sol–gel, microwave-assisted and wet chemical methods [20]. Johnston et al. proposed a chemical method to synthesize CS nanoparticles by the reaction of Ca ions with silicate anions at pH 11. CS nanoparticles can absorb metal particles or functional proteins to regulate immunological processes [21, 22]. In addition, CS nanowires were successfully fabricated via a hydrothermal method and have been proven to promote the adhesion, proliferation and osteogenesis of BMSCs in vitro [23, 24]. To improve the bioactivity of metal implants, CS coatings were added to a titanium alloy, and the nanostructure of the CS coating was further fabricated through a hydrothermal method, resulting in superior osseointegration capability [25–27]. Nevertheless, the direct construction of nanostructures on the surfaces of CS bioceramics still presented substantial challenges because of the brittleness of bioceramics.

Herein, flower-like nanostructures were first constructed in situ on the surface of CS bioceramics based on a facile hydrothermal treatment method without any surfactant or template. Subsequently, the flower-like nanostructures significantly promoted cell adhesion, proliferation and osteogenic differentiation. To further investigate the role of nanostructures in bone repair and regeneration in vivo*,* porous CS scaffolds were prepared via the porogen method, and then, nanostructures were generated in situ on CS scaffolds as described above. Ultimately, CS bioceramics with nanostructures exhibited strong abilities to promote bone regeneration in vivo. Based on all the results, nanostructure CS bioceramics could achieve the repair of bone defects, and these bioceramics could be candidates for use as bone implants in the clinic.

## Materials and methods

### Fabrication of nanostructure CS (nCS) bioceramics

CS powders were obtained from Kunshan Chinese Technology New Materials Co., Ltd. (China). The CS powders were mixed with 6 wt% polyvinyl alcohol (PVA), further compacted into discs under a pressure of 7 MPa in a stainless-steel die, and then heated at 1100 °C for 5 h to fabricate CS bioceramic discs with a diameter of 10 mm and a thickness of 2 mm. Subsequently, the CS discs were subjected to hydrothermal treatment (incubation in aqueous solutions with pH = 7 for 72 h at 180 °C) to generate flower-like nanostructure surfaces. The CS discs with nanostructure surfaces were labeled nCS and used in in vitro experiments.

To better observe bone regeneration in the animal experiments, porous CS bioceramic scaffolds were fabricated by the porogen method as described in a previous study [28]. Polyethylene glycol (PEG) with a diameter ranging from 300 to 600 μm was used as a porogen and mixed with CS powder in a suitable ratio with 6 wt% PVA. The mixture was pressed into a three-dimensional (3D) cylinder with a diameter of 5 mm and a thickness of 1 mm, and then, porous scaffolds were obtained after sintering at 1100 °C. Finally, CS bioceramic scaffolds with nanostructure surfaces were prepared after hydrothermal treatment and used in in vivo experiments.

CS bioceramic discs and scaffolds that were not subjected to hydrothermal treatment were considered control samples.

### Characterization and protein adsorption performance of nCS bioceramics

The crystal phases of the CS and nCS discs were characterized by X-ray diffraction analysis (XRD, Rigaku, Japan), Fourier-transform infrared spectrometry (FTIR, Nicolet iS 10, Thermo, USA), and X-ray photoelectron spectroscopy (XPS, Thermo Kalpha, USA). The surface morphology of CS bioceramics was characterized by field emission scanning electron microscopy (FESEM, JEOL, Japan), transmission electron microscopy (TEM, FEI Tecnai F20, Japan), scanning transmission electron microscopy (STEM, FEI Tecnai F20, Japan) and energy dispersive spectrometer (EDS).

To evaluate the biodegradability of bioceramics, the CS and nCS discs were immersed in Tris–HCl buffer (pH = 7.4, 37 °C) at a ratio of 0.1 (mm^3^:ml), and the solution was refreshed every 2 days. At 1, 3, 7, 10, and 14 days, the pH value of Tris–HCl was measured with a pH meter (Mettler Toledo, Switzerland), and the concentrations of Ca and Si ions were measured by inductively coupled plasma atomic emission spectroscopy (ICP-AES, Varian, USA) [29]. In addition, the ions release of CS and nCS bioceramics in phosphate buffer solution (PBS, pH = 7.4, 37 °C, Gibco, USA) for 14 days also was measured as described above. After 14 days, the surface of CS bioceramics was observed by SEM–EDS mapping. The wetting of the CS and nCS samples was analyzed by a goniometer (SZ-CAM, SUNZERN, Shanghai, China). In brief, the CS and nCS samples were placed on the sample stage, and 10 μl Milli-Q water (Millipore, water purification system model Direct-Q 3 UV, Merck, USA) was added to each sample (n = 5) via syringe by a water dispensing system. The contact angle between the tangent to the liquid–vapor interface and the solid surface at the three-phase contact line was calculated by a computer system.

To assess the protein adsorption ability of CS and nCS, 15 mg/ml bovine serum albumin (BSA) solution was applied on both samples. After soaking both samples in 1 ml BSA solution (C0, 15 mg/ml) for 48 h at 37 ℃, the rest protein concentration (C1) was measured by Bicinchoninic Acid (BCA) Protein Assay Kit (Thermo Scientific TM, USA). The amount of BSA adsorption and the loading capacity of both samples were calculated according to the formulae below:

- Protein adsorption amount (mg) = (C0−C1) × V_solution_.
- Loading capacity (%) = (C0−C1)/C0 × 100%
- C0: represented the original concentration of BSA solution.
- C1: represented the rest concentration of BSA solution.

### Cell culture

Sprague–Dawley (SD) rats (2 weeks old) were purchased from Shanghai Sippr-BK Laboratory Animal Co. Ltd. (China). Bone marrow stem cells (BMSCs) were extracted from the femur and tibia of the rats as previously reported [30]. Then, the BMSCs were cultured in α-minimum essential medium (α-MEM, Gibco, USA) supplemented with 10% FBS (Gibco, USA) and 1% penicillin–streptomycin (Gibco, USA). The cultures were maintained in an incubator (37 °C, 5% CO_2_, and 95% relative humidity), and the medium was refreshed 3 times a week to remove the nonadherent cells. After reaching 80%–90% confluence, the BMSCs were passaged, and passages 2–4 were used in the in vitro experiments in the study. To decrease the alkalinity of CS bioceramics, we soaked all the samples in deionized water for two weeks and changed deionized water every day. After soaking in deionized water for two weeks, CS and nCS samples were directly used for subsequent in vitro and in vivo experiments.

### Cell adhesion and morphology

BMSCs were seeded on CS and nCS discs (diameter of 10 mm) at a density of 2 × 10^4^ cells per well in 24-well culture plates with 1.5 ml medium. Culture medium with CS and nCS bioceramics was also changed every day. After culturing for 6 h, the cells on the bioceramic discs were washed with PBS 3 times and fixed with 4% paraformaldehyde for 30 min according to the manufacturer’s instructions. Then, all the cells were incubated with phalloidin (Sigma, USA) for 30 min and 4′,6-diamidino-2-phenylindole (DAPI, Sigma, USA) for 5 min and then observed with a confocal laser scanning microscope (CLSM, Leica, Germany). At 24 h after incubation, the cells on CS and nCS bioceramics were fixed with 2.5% glutaraldehyde at 4 °C for 12 h. The samples were further fixed with 1% osmic acid solution for 2 h, dehydrated with gradient concentrations of ethanol solution (30%, 50%, 70%, 80%, 90% and 95%), coated with gold and finally observed by using scanning electron microscopy (SEM, JEOL, Japan).

Immunofluorescence staining was used to further evaluate the effect of nCS bioceramics with flower-like nanostructures on the expression of focal adhesion proteins in cells. BMSCs were seeded on CS and nCS bioceramics at a density of 2 × 10^4^ cells per well in 24-well culture plates with 1.5 ml medium. After being cultured for 6 h, the BMSCs were fixed with 4% paraformaldehyde for 30 min, rinsed with PBS 3 times and permeabilized with 0.3% Triton-X 100. Then, the cells were further blocked in 1% bovine serum albumin (BSA) for 1 h. Both groups were incubated with primary rabbit anti-rat vinculin monoclonal antibodies (1:200, Abcam, USA) overnight at 4 °C on a table shaker to measure the expression level of focal adhesion proteins. Then, the samples were incubated with Alexa Fluor 594-conjugated goat anti-rabbit IgG secondary antibodies (1:500, Thermo Fisher, USA) at 37 °C for 1 h. The cytoskeleton and cellular nuclei were stained with FITC-phalloidin and DAPI, respectively. Images were captured with an inverted fluorescence microscope (Olympus, Japan). All the experiments were repeated three times.

### Cell proliferation and biocompatibility in vitro of CS bioceramic

The MTT assay was carried out to investigate the proliferation of cells cultured on CS and nCS bioceramics (diameter of 10 mm); the BMSCs were cultured at an initial density of 1 × 10^4^ cells per well in 24-well culture plates in 1.5 ml culture medium, which was replaced every day. After 1, 3, and 7 days of culture, all the cells cultured on bioceramics were incubated with MTT solution (5 mg/ml) at 37 °C for 4 h. Subsequently, dimethyl sulfoxide (DMSO, Sigma, USA) solution was added, and the absorbance (OD value) was read at 490 nm with a microplate reader (Biotek, USA). All the experiments were repeated three times.

To evaluate the blood compatibility of CS bioceramics in vitro, a hemolytic test was used to evaluate CS and nCS samples as previous study [31]. Fresh blood cells (BCs) were extracted from 2-week-old SD rats purchased from Shanghai Sippr-BK Laboratory Animal Co. Ltd., and the BCs were centrifuged at 2000 rpm for 5 min. The 4% BC solutions were obtained by mixing 0.2 ml BCs with 4.8 ml PBS solution, and both samples were incubated in PBS for 24 h to obtain the sample solution. Then, 0.5 ml BC solution was incubated in 0.5 ml sample solution, Triton X-100 and PBS at 37 °C for 1 h. Triton X-100 was used as a positive control, and PBS was used as a negative control. The supernatants were harvested by centrifuging the samples at 2000 rpm for 5 min, and the supernatants were photographed with a digital camera. Ultimately, the OD value of the supernatants were measured with a microplate reader at a wavelength of 540 nm.

### Alkaline phosphate (ALP) activity and staining assay

BMSCs were seeded on CS and nCS discs at a density of 2 × 10^4^ cells per well in 24-well culture plates in 1.5 ml culture medium, which was replaced every day. At 4, 7 and 10 days of culture, the cells cultured on bioceramics were rinsed with PBS, lysed with 1% Triton X-100, and centrifuged at 4 °C, and ALP activity was quantified with an ALP kit (Beyotime, China) according to the manufacturer’s protocol. The absorbance was read at 405 nm with a spectrophotometer, and the total cellular protein content was determined using a BCA protein kit (Beyotime, China). The ALP activity was normalized to the total protein concentration. To intuitively characterize the effect of nanostructures on ALP expression, an ALP staining assay was also carried out using an ALP staining kit (Beyotime, China) at Days 4, 7 and 10. All the experiments were repeated three times.

### Quantitative real-time PCR (qRT–PCR) and immunofluorescence analyses of Runx2 expression

BMSCs were seeded on CS and nCS bioceramics (diameter of 25 mm) at a density of 1 × 10^5^ cells per well in 6-well culture plates in 5 ml medium, which was replaced every day. After being cultured for 7 days, total RNA was isolated from each well using TRIzol Reagent and then reverse transcribed to complementary DNA (cDNA) by a PrimeScript™ RT Kit (TaKaRa, Japan). The expression levels of bone sialoprotein (Bsp), runt-related transcription factor-2 (Runx2), collagen type I alpha 1 (Col1a1), and osteopontin (Opn) were quantified, and glyceraldehyde-3-phosphate-dehydrogenase (GAPDH) was used as a housekeeping gene for normalization. The primer sequences used in this study were presented in Additional file 1: Table S1.

BMSCs were seeded on both types of bioceramics (diameter of 10 mm) at a density of 1 × 10^4^ cells per well in 24-well plates in 1.5 ml medium and were cultured for 7 days. Cell fixation with 4% PFA, cell permeability with 0.3% Triton X-100, and cell blocking with 1% BSA were performed. Both groups were incubated with primary rabbit anti-rat Runx2 monoclonal antibodies (1:5000, Abcam, USA) overnight at 4 °C on a table shaker to measure the expression level of Runx2. Furthermore, cell cytoskeleton and cellular nuclei staining were performed as described above. Images were captured with an inverted fluorescence microscope.

### Western blotting experiments

Western blotting was used to assess the molecular mechanism underlying the effects of CS and nCS. The protein expression levels of focal adhesion kinase (FAK), phospho-focal adhesion kinase (p-FAK) and MAPK signaling pathway-related proteins (extracellular signal-related kinase, ERK; phospho-ERK, p-ERK; c-Jun-amino-terminal kinase, JNK; phospho-JNK, p-JNK; p38; phospho-p38, p-p38) were measured, and β-actin expression was used as a reference. BMSCs (3 × 10^5^) were cultured on CS and nCS bioceramics (diameter of 25 mm) in 6-well plates in 5 ml medium per well for 48 h. Additionally, the FAK signaling pathway inhibitor PF573228 (1 μM, Sigma, USA) and p38 signaling pathway inhibitor SB203580 (10 μM, MedChemExpress, USA) were separately added to the culture medium for further studies. Total protein was extracted with RIPA buffer (Beyotime, China) supplemented with 1% phenylmethanesulfonylfluoride (PMSF, Beyotime, China) at 4 ℃ for 5 min, and the samples were centrifuged at 12,000 rpm for 15 min. Then, the cellular supernatants were collected, and the protein concentrations were quantified with a BCA protein assay kit (Thermo Fisher, USA). Protein samples were separated via sodium dodecyl sulfate–polyacrylamide gel electrophoresis (SDS–PAGE) at 80 V for 20 min and 120 V for 50 min, and then, the separated proteins were transferred to polyvinylidene fluoride membranes (PVDF, Millipore, USA). After blocking with 5% skim milk, the PVDF membranes were incubated with each primary antibody (Abcam, UK) overnight at 4 °C. Then, the PVDF membranes were washed three times with Tris-buffer solution with Tween (TBST, Beyotime, China) and incubated with HRP-conjugated secondary antibodies (Abcam, UK) for 1 h at room temperature. The protein bands were visualized, and images were captured with an automated luminescent image analysis system (Tanon, China).

### Animal procedures and evaluation of bone regeneration

Six-week-old SD rats were purchased from Shanghai Sippr-BK Laboratory Animal Co., Ltd. to establish calvarial defects. Subsequent animal experiments were approved by the Institutional Animal Care and Use Committee (IACUC) of Shanghai Ninth People's Hospital Affiliated with Shanghai Jiaotong University, School of Medicine. All the rats were randomly divided into 3 groups. A 2-cm longitudinal incision was made after general anesthesia was achieved via the intraperitoneal injection of 3.5% pentobarbital sodium. The scalp was separated for exposition, and two symmetrical round defects 5 mm in diameter were made at the frontal bone of each rat using a drill. To avoid individual differences in the experiments, eighteen calvarial defects were constructed in nine SD rats. Three experimental modalities were randomly allocated to eighteen defects (n = 6), as follows: 1) blank control; 2) CS group; 3) nCS group. At 2, 4, and 6 weeks after the operation, all the rats were intraperitoneally injected with tetracycline hydrochloride (TE, 25 mg/kg), alizarin red (AL, 30 mg/kg), and calcein (CA, 20 mg/kg) to assess new bone formation and mineralization.

After being implanted for 8 weeks, the specimens (bone and scaffolds) were harvested, fixed in 4% paraformaldehyde for 3 days and then scanned with microcomputed tomography (PerkinElemer, QuantumGX, Japan) to analyze the bone volume fraction (BV/TV) in the defect area with auxiliary software (Analyze 12.0, Japan). On the one hand, half of the samples from all the groups were dehydrated with ascending concentrations of ethanol solution (75%–100%), embedded in polymethylmethacrylate (PMMA), sectioned using a microtome and further ground to a final thickness of 50 μm. Subsequently, fluorescent labeling of the samples was observed with CLSM (Leica, Germany), and the area of fluorochrome-stained bone and the distance between fluorescent stripes were measured with ImageJ (NIH; http://rsb.info.nih.gov/ij, USA). Next, the sections were stained with Van Gieson’s (VG) to observe the formation of new bone. The area of new bone formation in the VG-stained sections was measured with ImageJ. On the other hand, half of the samples from all the groups were decalcified with 10% EDTA for 1 month, dehydrated, embedded in paraffin, and stained using hematoxylin and eosin (H&E) and Masson’s trichrome staining [32–34].

To evaluate the biosafety in vivo of the CS bioceramics, CS and nCS bioceramics were implanted into the bilateral pockets of the perivertebral fascia lumbodraslis of SD rats under anesthesia (0.5 mg/kg of pentobarbital sodium). Briefly, the wounds were irrigated with normal saline and sutured in layers, and the animals were euthanized by carbon dioxide (CO_2_) asphyxia after 2 weeks. The implanted bioceramics and neighboring tissues were harvested for digital images. Subsequently, CS and nCS were removed and the subcutaneous tissue (Skin) was performed histological analysis, including H&E and immunohistochemical staining of tumor necrosis factor-a (TNF-α).

### Statistical analysis

All the data are presented as the mean ± standard deviation (SD). Statistical analysis was performed by one-way analysis of variance using SPSS 22.0 software (IBM Inc., USA). A p value less than 0.05 was considered statistically significant.

## Results and discussion

### Characterization of nCS bioceramics

**Fig. 1:**
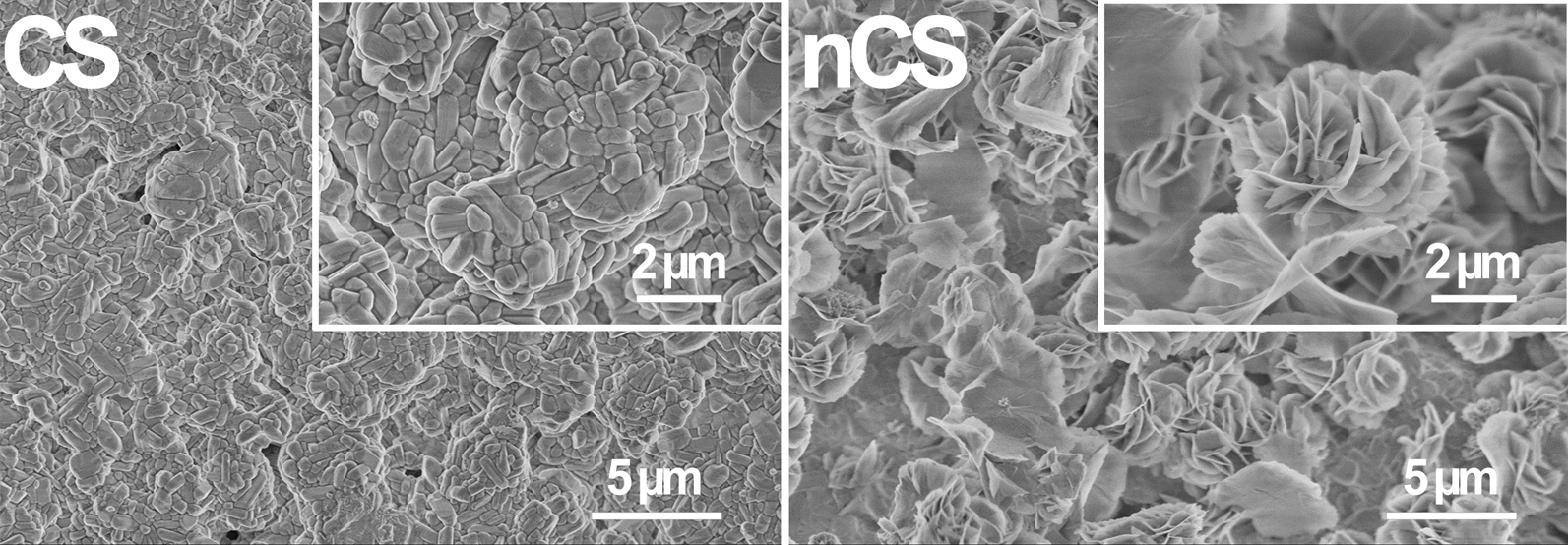
FESEM images of the surface topography of CS and nCS bioceramic discs (Scale bar = 5 μm at low magnification; scale bar = 2 μm at high magnification)

As shown in Fig. 1, CS bioceramic discs exhibited flat surfaces and short rod-like grains with dense arrangements. In comparison, significant amounts of flower-like nanostructures with thicknesses of 20–50 nm were observed on the surface of the nCS bioceramic discs. The flower-like nanostructures provided more sites for cellular adhesion, spreading and proliferation. Additional file 1: Fig. S1 showed that CS scaffolds prepared by the porogen method presented a porous structure with a diameter of 100–500 μm. In addition, the porous structure of nCS scaffolds was similar to that of CS scaffolds, and this structure was not changed by hydrothermal treatment, which could induce BMSCs to adhere and proliferate easily among the pore structures [35].

**Fig. 2.**
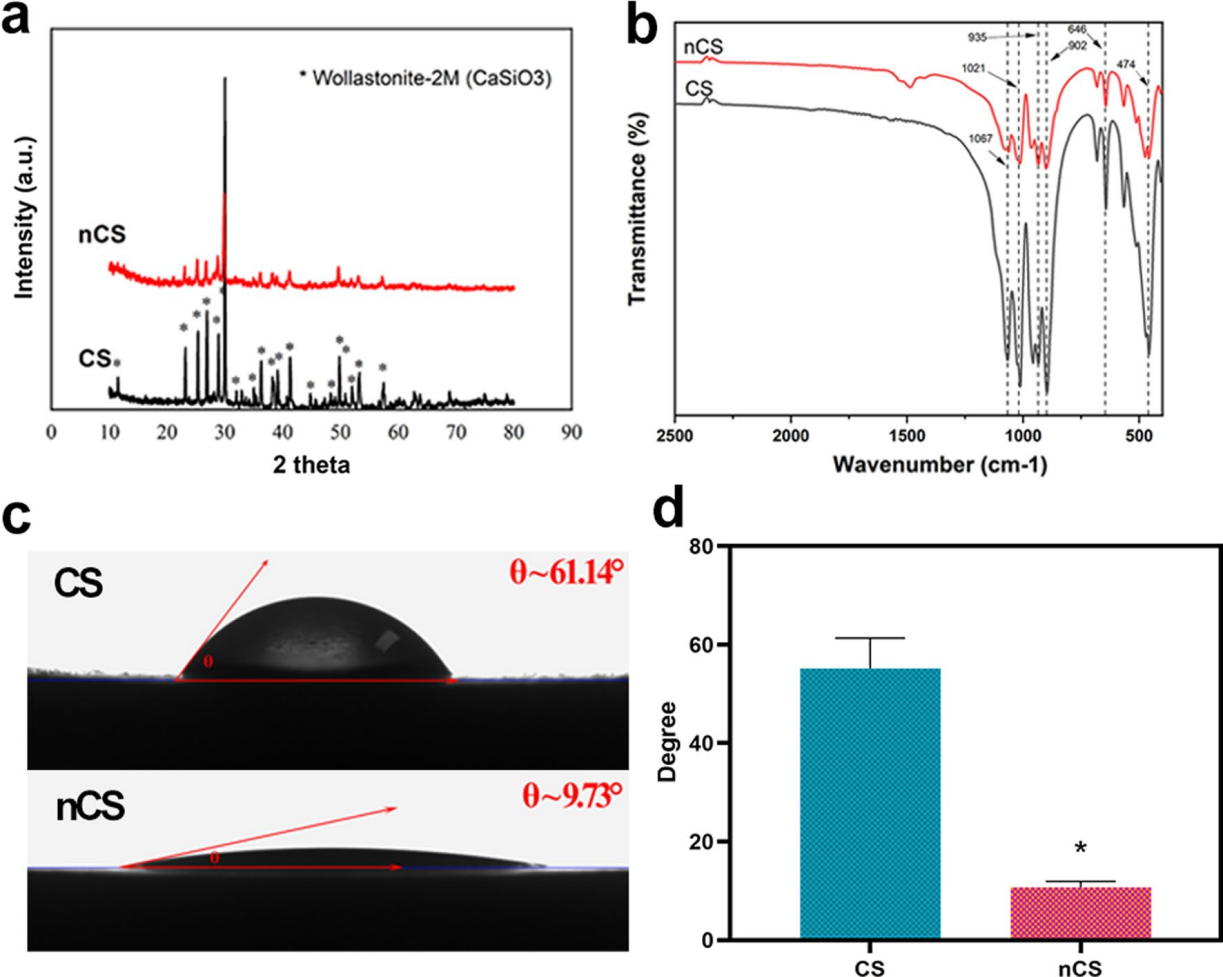
**a** XRD patterns of CS and nCS discs. **b** FTIR spectra of CS and nCS discs. **c** Intuitive optical pictures of the contact angles of CS and nCS bioceramic discs and **d** quantitative analysis. (*indicates significant differences, p < 0.05)

TEM and STEM with mapping were performed to examine the surface of nCS bioceramics (Additional file 1: Fig. S2). The result showed that the surface of nCS bioceramics presented the nano-sheet structure (Additional file 1: Fig. S2a, b), which could be explained by the fact that the flower-like structure composed of nano-sheets was ultrasonic dispersed into nanosheets during the test preparation process for TEM and STEM. In addition, as shown in EDS-mapping (Additional file 1: Fig. S2c–e), Si, O, and Ca elements were observed in the nanosheets of nCS. The further chemical composition analysis of nano-sheets from nCS bioceramics have been identified as calcium silicate, which was consistent with the result of SEM. Figure 2a showed that the main phases of the fabricated CS and nCS discs were wollastonite-2 M (CaSiO3, JCPDS card: No. 75–1396). This result suggested that the hydrothermal treatment (HT) did not affect the surface phase composition. FTIR analysis also was performed on the samples to further confirm the ingredients (Fig. 2b). There were no differences detected between CS and nCS bioceramics, and strong transmittance peaks at 474, 646, 902, 935 and 1067 cm^−1^ could be observed in FTIR spectra. The band at 474 cm^−1^ was attributed to ring structure of SiO_4_ tetrahedral, the band at 646 cm^−1^ was due to Si–O-Si symmetric stretching vibrations, and the bands at 902 and 935 cm^−1^ were characteristic peaks of Si–O-Ca. In addition, the band at 1067 cm^−1^ was due to stretching modes of Si–O–Si bond and the band at 935 cm^−1^ was related to Si–OH stretching, being consistent with the peaks of the calcium silicate. In addition, XPS also was applied to measure nCS bioceramics (Additional file 1: Fig. S3), confirming the presence of calcium (Ca), silicon (Si), and oxygen (O) elements. The presence of carbon (C) element at 284.8 eV binding energy was because of the adsorption of C element from the air. XPS O 1 s spectra reflected three chemical bond binding modes, which were Ca–O, Si–O and Si–O/C_2_O (Additional file 1: Fig. S3b, f). XPS Si 2p spectra indicated two species chemical bonds of Si elements, corresponding to Si–O and SiO_2_ (Additional file 1: Fig. S3c, g). Moreover, XPS Ca 2p1 and Ca 2p3 peaks were composed of CaO and CaCO_3_ (Additional file 1: Fig. S3d, h). As shown in Additional file 1: Table S2, the contents of elements also were measured by XPS. The result exhibited that the molar ratio of Ca and Si and O was about 1:1:3 in CS and nCS bioceramics, which suggested that the flower-like nanostructure on surface of nCS bioceramics was calcium silicate. Thus, all results indicated that calcium silicate with flower-like nanostructure was constructed in situ on CS bioceramic substrates. As shown in Fig. 2c, d, nCS bioceramics with flower-like nanostructure surfaces exhibited a smaller contact angle and showed better hydrophilicity than CS bioceramics. The wettability of biomaterials is important for cell-biomaterial contact and is beneficial for cell adhesion [36]; the results revealed that nCS bioceramics with flower-like nanostructures could promote cell adhesion, possibly by adsorbing ECM proteins to mediate cell–matrix anchorage, and further enhance osteogenic differentiation and tissue regeneration [37]. Moreover, the protein adsorption of nCS was 6.695 mg per sample, while CS was 3.776 mg per sample (Additional file 1: Fig. S7a). In addition, nCS showed stronger loading capacity (45.3%) than that of CS (25.18%) due to the better hydrophilicity on flower-like nanostructured surface (Additional file 1: Fig. S7b), indicating the remarkable protein loading capacity of nCS. Preferable protein adsorption capacity was beneficial for the formation of focal adhesions, which was conducive to osteogenesis and vascularization [38].

**Fig. 3:**
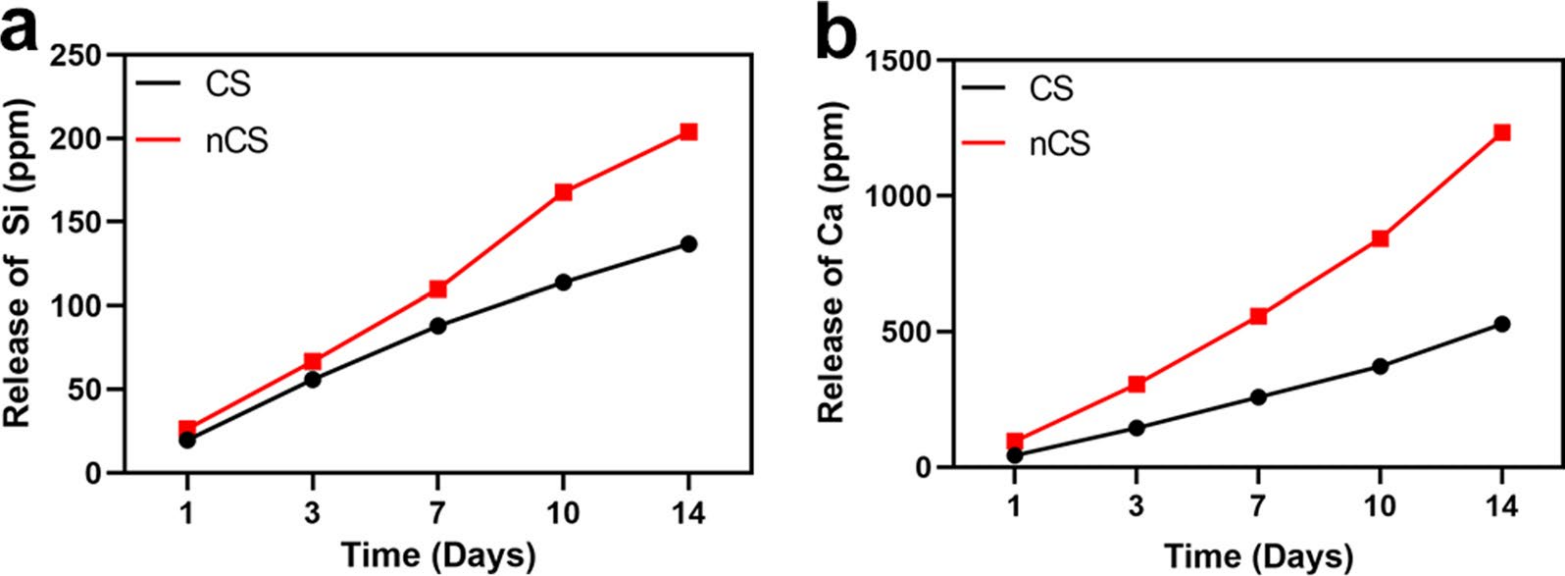
The release curve of Si (**a**) and Ca (**b**) ions from CS and nCS bioceramics in Tris–HCl for 14 days

The biodegradability of bone repair biomaterials was an important factor to consider. In this study, the amount of calcium (Ca) and silicon (Si) ions released was calculated to evaluate the biodegradability of nCS bioceramics by soaking CS and nCS bioceramics in Tris–HCl buffer solution and PBS solution, respectively. As shown in Fig. 3, the release of Ca ions and Si ions from nCS bioceramics was slightly higher than that from CS bioceramics due to the reduction in the crystallization of grains on the nCS surface after hydrothermal treatment [39]. As shown in Additional file 1: Fig. S4, the pH value of Tris–HCl solution with nCS bioceramics for 14 days was higher than that with CS bioceramics. Previous study showed that microenvironment with alkalescence was critical for biomaterials to promote tissue regeneration because slight alkalinity could neutralize acidic metabolites around bone defects and further facilitate osteogenesis [40]. Based on the results, we believed that nCS bioceramics possessed favorable osteogenic ability. In addition, we performed an experiment of the ions release in PBS solution (Additional file 1: Fig. S5) as a comparison. As compared with the result of ions release in Tris–HCl, the release of Ca and Si elements in PBS solution from CS and nCS bioceramics was significantly lower. To explain the phenomenon, SEM–EDS was employed to examine the surface morphology and chemical composition of CS bioceramics immersed into PBS solution for 14 days (Additional file 1: Fig. S6). As shown in Additional file 1: Fig. S6, calcium phosphate was generated on the surface of CS bioceramics without hydrothermal treatment. The results indicated that in addition to degradation, biological mineralization took place when CS bioceramics were soaked in PBS solution, and thus reduced the ions release rate. Considering that silicon ions have been shown to promote angiogenesis and bone regeneration [41], there was a good reason to believe that nCS bioceramics could facilitate cell behaviors, including adhesion and osteogenic differentiation.

### Cell adhesion, spreading, proliferation and blood compatibility analyses

**Fig. 4:**
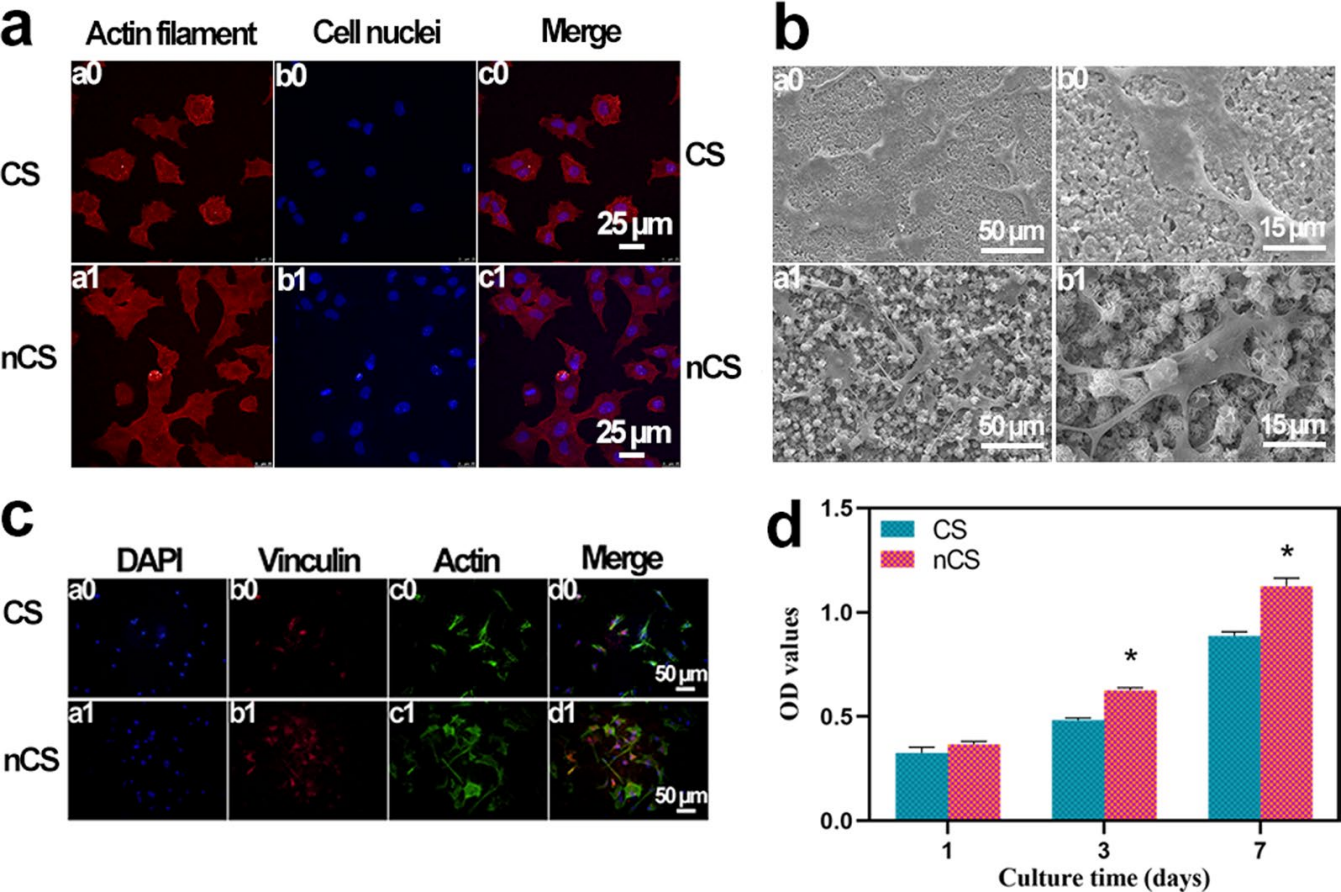
Evaluation of the adhesion, spread, and proliferation of BMSCs cultured on CS and nCS bioceramics. **a** CLSM images of cells cultured for 6 h. Red: actin cytoskeleton (a0, a1), blue: cell nuclei (b0, b1), merge: actin cytoskeleton and cell nuclei (c0, c1). Scale bar = 25 μm. **b** SEM images of cells cultured for 24 h. (a0, a1) Scale bar = 50 μm; (b0, b1) scale bar = 15 μm; **c** Immunofluorescence images of BMSCs cultured for 6 h: (a0, a1) cell nuclei were stained blue, (b0, b1) vinculin was stained red, (c0, c1) the actin was stained green, (d0, d1) merged image of the cell nuclei, vinculin, and the cytoskeleton. Scale bar = 50 μm. **d** MTT quantitative analysis of cells cultured for 1, 3, and 7 days. (*indicates significant differences, p < 0.05)

Nanostructures have been proven to stimulate protein adsorption and further enhance cell adhesion and proliferation through the embedding of filopodia in the sample surfaces. In this study, the adhesion and morphology of BMSCs cultured on bioceramics was observed, as shown in Fig. 4a. The cells cultured on nCS bioceramics exhibited a large spread, while the cells cultured on CS bioceramics displayed a round shape and exhibited a small spread. The cellular morphologies were further evaluated by SEM. As shown in Fig. 4b, the results showed that cells cultured on nCS bioceramics spread well and exhibited longer filopodia than those cultured on CS bioceramics, which was consistent with the results in Fig. 4a. Focal adhesions (FAs), which contain adhesion-related proteins, can provide structural connections between cells and the extracellular matrix (ECM) and play important roles in regulating cell morphology, growth, proliferation and differentiation [42]. In this study, the expression of the vinculin protein, one of the most abundant FA proteins, was measured with immunofluorescence staining. As shown in Fig. 4c, the expression of vinculin in the cells cultured on nCS bioceramics was higher than that in the cells cultured on CS bioceramics, revealing that flower-like nanostructures could promote cell adhesion and possessed the potential to accelerate osteogenic differentiation. Compared with Triton-X 100 group, the suspension in PBS group, CS group and nCS group was colorless and clarified (Additional file 1: Fig. S8a). In addition, the relevant quantitative analysis also further exhibited that CS and nCS bioceramics possessed good blood compatibility and biosafety in vitro (Additional file 1: Fig. S8b).

Generally, nanostructured surfaces not only promoted cellular attachment and spread but also accelerated proliferation [43]. As shown in Fig. 4d, the BMSCs in both groups proliferated well during the whole experimental period, indicating the excellent biocompatibility of CS and nCS bioceramics. Furthermore, the cells cultured on nCS bioceramics proliferated better than those cultured on CS bioceramics, and significant differences were observed after culturing the cells for 3 and 7 days (p < 0.05), which indicated that the flower-like nanostructure surface of nCS bioceramics has the capacity to promote the proliferation of BMSCs.

### ALP activity and staining assay

**Fig. 5:**
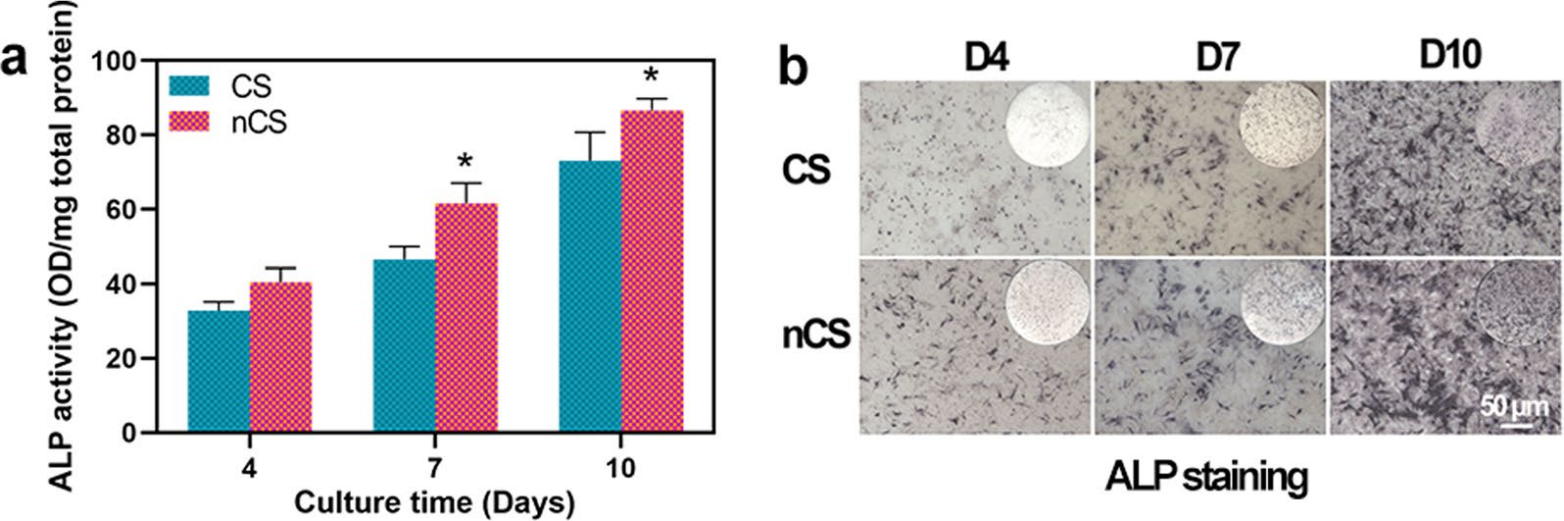
ALP activity of BMSCs cultured on CS and nCS bioceramics. **a** Measurement of the ALP activity of cells cultured on bioceramics for 4, 7, and 10 days. **b** ALP staining of cells cultured on bioceramics for 4, 7, and 10 days. (* indicates significant differences, p < 0.05)

ALP was a marker of osteogenesis that could reflect the activity and differentiation level of osteoblasts. At the stage of bone formation and extracellular calcium salt sedimentation, the expression of ALP was high [44]. The results in Fig. 5a suggested that the ALP activity of the cells cultured on nCS bioceramics was higher than that of the cells cultured on CS bioceramics, and statistically significant differences were observed at 7 and 10 days (p < 0.05), which indicated the better effect of flower-like nanostructures on promoting osteogenesis. Furthermore, an ALP staining assay was performed to assess ALP activity. As shown in Fig. 5b, the cells cultured on nCS bioceramics exhibited more intense ALP staining throughout the whole experimental period than the cells cultured on CS bioceramics. A recent study confirmed that nanotopographies could induce osteogenic differentiation, which was consistent with our results, further suggesting the role of flower-like nanostructures in promoting bone regeneration [45, 46].

### Quantitative real-time PCR (qRT–PCR) and immunofluorescence analyses of Runx2 expression

**Fig. 6.**
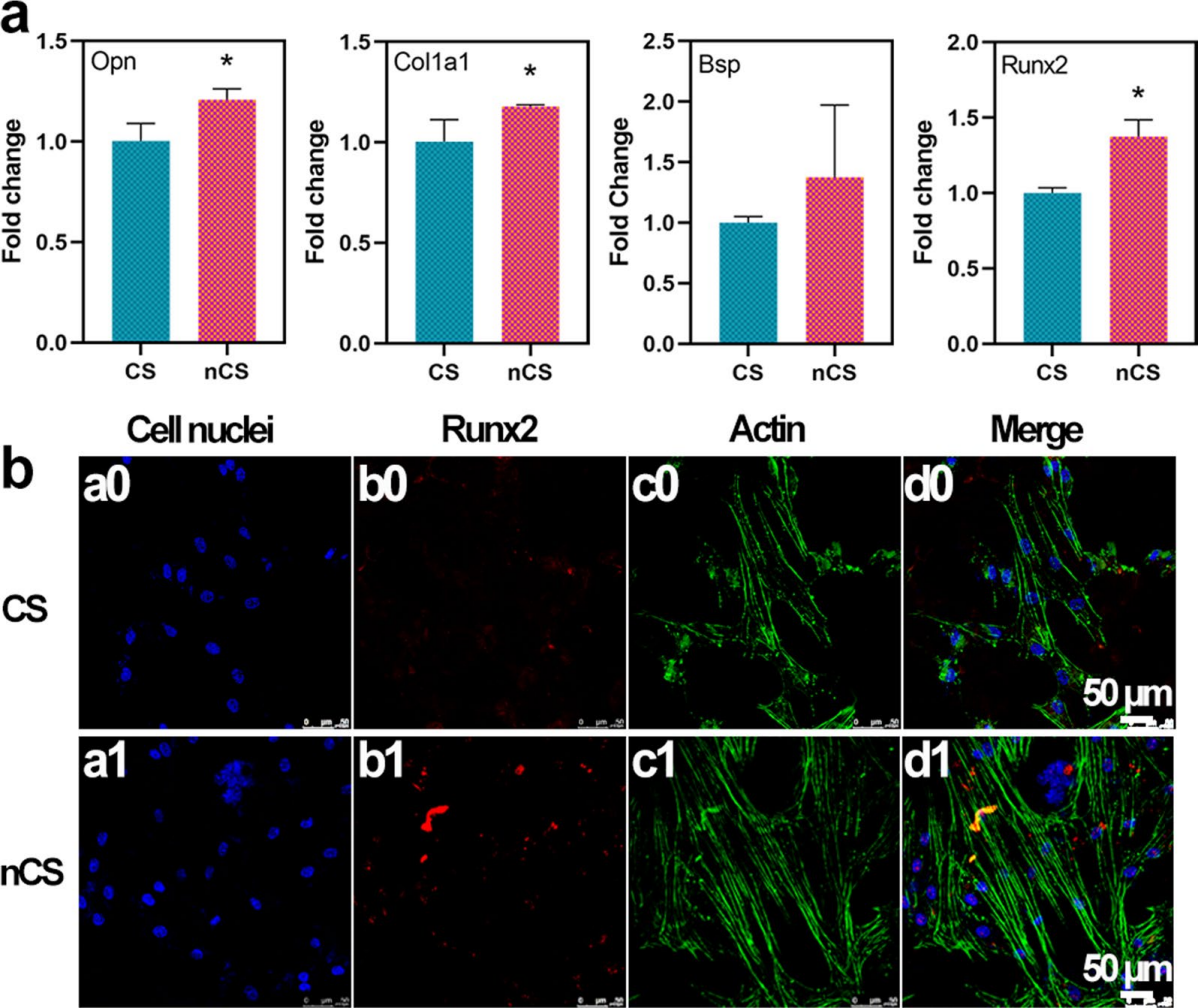
**a** The mRNA expression levels of Opn, Col1a1, Bsp and Runx2 in BMSCs cultured on CS and nCS bioceramic discs for 7 days. (* indicates significant differences, p < 0.05). **b** The immunofluorescence staining of Runx2 in CS and nCS bioceramic discs for 7 days. CLSM images of the cells cultured for 7 days. Blue: the cell nuclei (a0, a1), red: Runx2 (b0, b1), green: the actin (c0, c1), merge: Runx2, actin and cell nuclei (d0, d1). Scale bar = 50 μm

The differentiation of mesenchymal cells into osteoblasts was the initial step of bone formation, and this process was regulated by several osteogenic marker genes, including Runt-related transcription factor-2 (Runx2), Osteopontin (Opn), Collagen Type I Alpha 1 (Col1a1), and Bone sialoprotein (Bsp). Runx2 encoded an osteoblast-specific transcription factor that regulated osteoblast differentiation and bone formation [47]. Opn was an important gene that controls the formation of the bone matrix, and Col1a1 was an extracellular matrix structural component. Bsp was an osteogenic marker associated with the biomineralization of collagen [48]. A qRT–PCR assay was performed to measure the expression of genes involved in osteogenesis in BMSCs cultured on CS and nCS. As shown in Fig. 6a, the expression of the target genes was higher in cells cultured on nCS bioceramics than in cells cultured on CS bioceramics, and significant differences in the expression of Runx2, Opn, and Col1a1 were further observed.

The immunostaining of Runx2 protein also was further performed for confirming the osteogenic ability of nCS bioceramics (Fig. 6b). As compared to the protein expression on CS bioceramics, Runx2 protein (red) showed higher expression level on nCS bioceramics, showing significant enhancement of flower-like nanostructure on osteogenesis differentiation. Previous studies have suggested that silicon-based bioceramics could promote the osteogenic differentiation without any osteogenic reagents such as growth factors, which was because of the bioactive silicon ions released from bioceramics could stimulate osteogenesis via activating BMP2 signaling pathway [13, 49]. Our results confirmed that nanostructured surface modification could significantly promote osteogenic differentiation without any induction of osteogenesis, suggesting the outstanding osteogenic ability of flower-like nanostructures on nCS bioceramics.

### The impact of nanostructured CS on the FAK/p38 signaling pathway

**Fig. 7:**
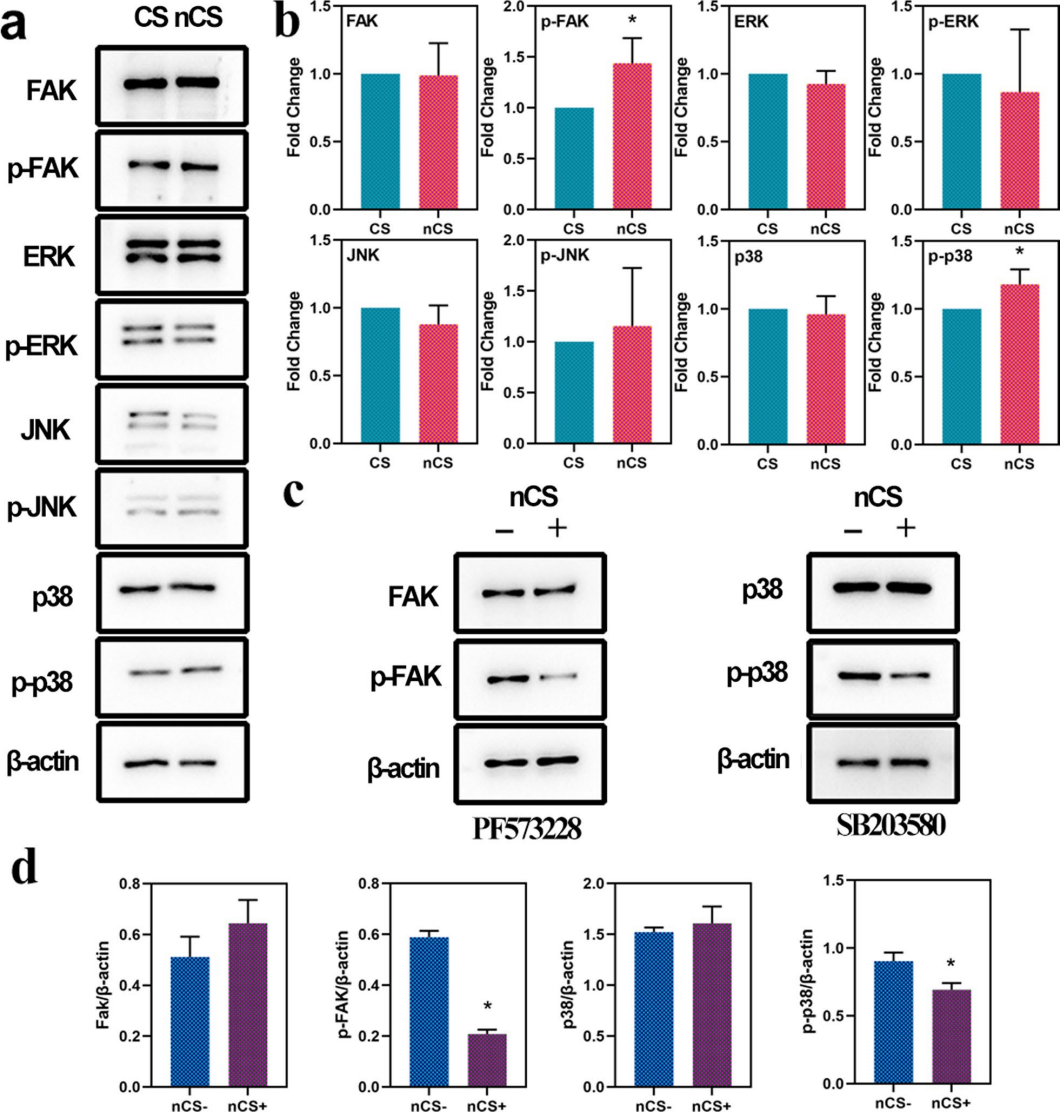
Western blotting analysis of the expression of FAK and MAPK signaling pathway-related proteins in vitro. **a** The expression of FAK, p-FAK and MAPK signaling pathway-related proteins in BMSCs cultured on CS and nCS bioceramics for 48 h. **b** Quantitative analysis of the expression of FAK, p-FAK and MAPK signaling pathway-related proteins (*p < 0.05). **c** The expression of FAK, p-FAK and MAPK signaling pathway-related proteins in BMSCs cultured on CS and nCS bioceramics in the presence of FAK and p38 inhibitors. **d** Quantitative analysis of the expression of FAK, p-FAK and MAPK signaling pathway-related proteins in cells treated with FAK and p38 inhibitors. (*p < 0.05)

FAK was a type of cytosolic nonreceptor protein-tyrosine kinase (PTK) that regulated the growth, proliferation and differentiation of cells [50], and FAK played a vital role in modulating various downstream intracellular signaling pathways, such as the MAPK signaling pathway [51]. The MAPK family included at least ERK, JNK/SAPK and p38 MAPK, which participated in the regulation of drivers of cell cycle progression and different kinds of complex cellular programs [52, 53]. The expression levels of FAK, p-FAK and MAPK signaling pathway-related proteins (ERK, JNK, p38) were investigated by western blotting. As shown in Fig. 7a, b, the expression level of p-FAK in the nCS group was higher than that in the CS group. nCS bioceramics with flower-like nanostructures promoted higher expression of phosphorylated p38 than CS bioceramics. However, no difference was observed in the expression of FAK, ERK, p-ERK, p38, JNK and p-JNK between the two groups. In addition, the results in Fig. 7c, d demonstrated that the expression levels of p-FAK and p-p38 in the nCS group were both decreased in the presence of specific inhibitors, as demonstrated by Western blotting and the subsequent quantitative analysis, indicating that flower-like nanostructures could activate p-FAK and furthermore activated p-p38, which played a crucial role in osteogenesis [54]. Considering these results together with the qRT–PCR results, we believe that flower-like nanostructures could enhance cell adhesion and osteogenic differentiation mainly by activating the FAK/p38 signaling pathway.

### Evaluation of the ability of nCS bioceramics with flower-like nanostructures to promote bone regeneration in vivo

**Fig. 8:**
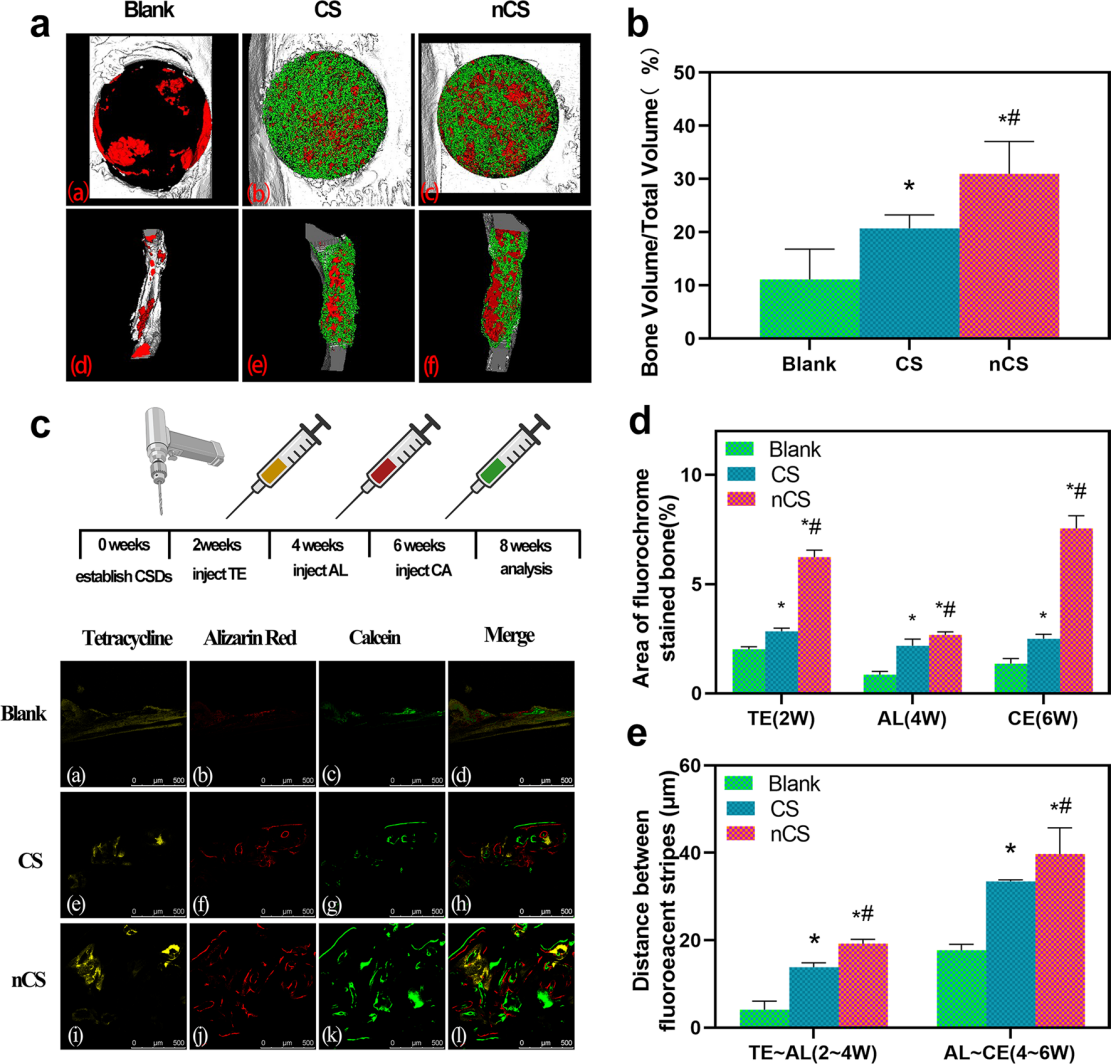
Micro-CT measurement and triple fluorescent labeling analysis of bone regeneration. **a** 3D and cross-sectional micro-CT images of the defect area in the 8^th^ week (red: newly formed bone, green: bioceramic scaffolds). **b** Quantitative analysis of the BV/TV ratio in the implantation region in the 8^th^ week. **c** Sequential fluorescent labeling images at 2, 4, and 6 weeks after the operation. **d** Quantitative evaluation of the area of stained bone. **e** Quantitative evaluation of the distance between fluorescent stripes. (*indicates a significant difference compared with the blank group, ^#^ indicates a significant difference compared with the CS group, P < 0.05)

CS and nCS bioceramics were implanted into critical-sized defects in rats to further evaluate their ability to promote bone regeneration. As shown in Fig. 8a, both CS and nCS bioceramics promoted the growth of more new bone toward the inside of the scaffolds compared with the Blank. Moreover, nCS bioceramics exhibited the strongest osteogenic ability, which was further proven by micro-CT quantitative analysis (Fig. 8b). The BV/TV values of the CS and nCS groups were larger than that of the control group (11.12%), and a remarkable BV/TV ratio (30.98%) was observed in the nCS group. A significant difference was observed between the CS and nCS groups (p < 0.05).

Subsequently, sequential fluorescent labeling was performed to evaluate bone formation and mineralization at different stages. As shown in Fig. 8c, the areas stained yellow, red, and green with different calcium-bonded dyes represented bone regeneration and remodeling at 2, 4, and 6 weeks, respectively. The results showed that the three groups exhibited abundant ossification at week 6 (Fig. 8c, CE, green). Compared with the Blank and CS groups, the nCS group displayed a larger stained bone area. In addition, the stripe distances between AL and CE also showed more bone formation in the nCS group in the 6th week than in the other two groups. Furthermore, Fig. 8d, and e showed the results of the quantification of the stained bone area, indicating that nCS bioceramics promoted bone formation and mineralization at every period of the experiment.

**Fig. 9:**
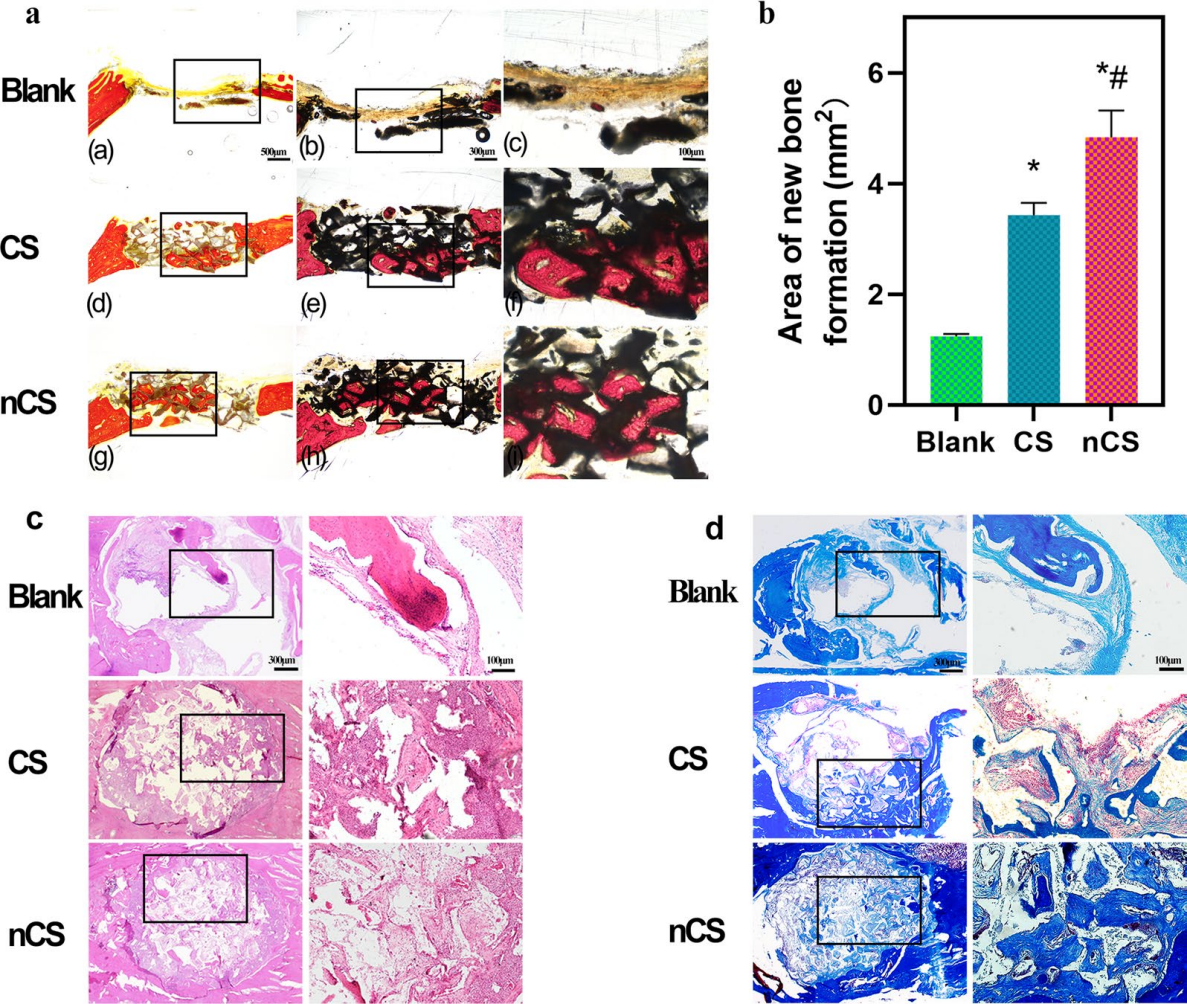
Histological staining analysis of bone regeneration. **a** Images of new bone stained with Van Gieson’s picrofuchsin and **b** related quantitative analysis. Histological images of bone tissue sections stained with H&E **c** and Masson trichrome **d**. (*indicates a significant difference compared with the blank group, ^#^indicates a significant difference compared with the CS group, P < 0.05)

Histological staining and statistical analysis provided more information about newly formed bone, and the results showed more regenerated bone tissue in the nCS group than in the control group. As shown in Fig. 9a, the bone tissues that were stained red almost filled the pore structures of the bioceramic scaffold, which was consistent with the micro-CT images (Fig. 8a). Further quantitative results (Fig. 9b) indicated that the nCS group exhibited a larger area of bone regeneration (4.83 mm^2^) than the other two groups. In addition, osteogenesis and osteointegration were assessed by H&E and Masson’s staining, as shown in Fig. 9c and d. In the nCS group, a greater area of blue staining was observed, indicating greater maturity of newly formed bone and excellent ability of the nCS scaffolds to promote bone regeneration. In this research, all the animals survived at the end of the experiment, and no wound infection was observed.

To test the biosafety of CS bioceramics in vivo, CS and nCS bioceramics were implanted in SD rat subcutaneous sites for two weeks. Blank group was regarded as negative control. The host tissue responses to the bioceramics were assessed by histomorphology analysis and immunohistochemical staining of tumor necrosis factor-α (TNF-α) as shown in Additional file 1: Figs. S9 and S10. As Additional file 1: Fig. S9 shown, no hyperemia and edema were observed between implant materials and surrounding tissues in blank, CS and nCS groups, and there were no pathological changes in the epidermis and dermis as well in blank, CS and nCS groups. As compared with blank group, H&E staining (Additional file 1: Fig. S10a1–a15) of skin which was removed bioceramics and important organs (Heart, Kidney, Liver, and Lung) in CS and nCS groups exhibited that no obvious morphological changes, neutrophil or lymphocyte infiltration and necrotic cells were observed in these tissues. TNF-α was one kind of biomarker involved in immunological regulation, infection and inflammation. There was no obvious expression of TNF-α in the junction of subcutaneous tissue (Skin) and important organs of all groups (Additional file 1: Fig. S10b1–b15), suggesting the biocompatibility and biosafety of CS and nCS bioceramics.

Previous studies have reported that surface modification could improve the osteogenesis performance of biomaterials. For instance, biomaterials modified with black phosphorus (BP), HA and SiO_2_ were successfully fabricated by utilizing microfluidic technology and showed considerable osteogenic ability [55]. However, HA and SiO_2_ were hard to degrade, especially the biosafety of SiO_2_ need to be further evaluated. To address the issue, we constructed the nanostructure CS bioceramics with favorable biodegradability on CS substrates. The nanostructure CS bioceramics could be completely taken place by new bone after being implanted in vivo.

In addition, some reports showed nanomaterials as delivery vehicles have been used for promoting bone formation via delivering drugs or bioactive molecules. In our work, flower-like nanostructure exhibited good protein loading capacity, which provided a promising potential to be regarded as nanocarriers.

**Fig. 10:**
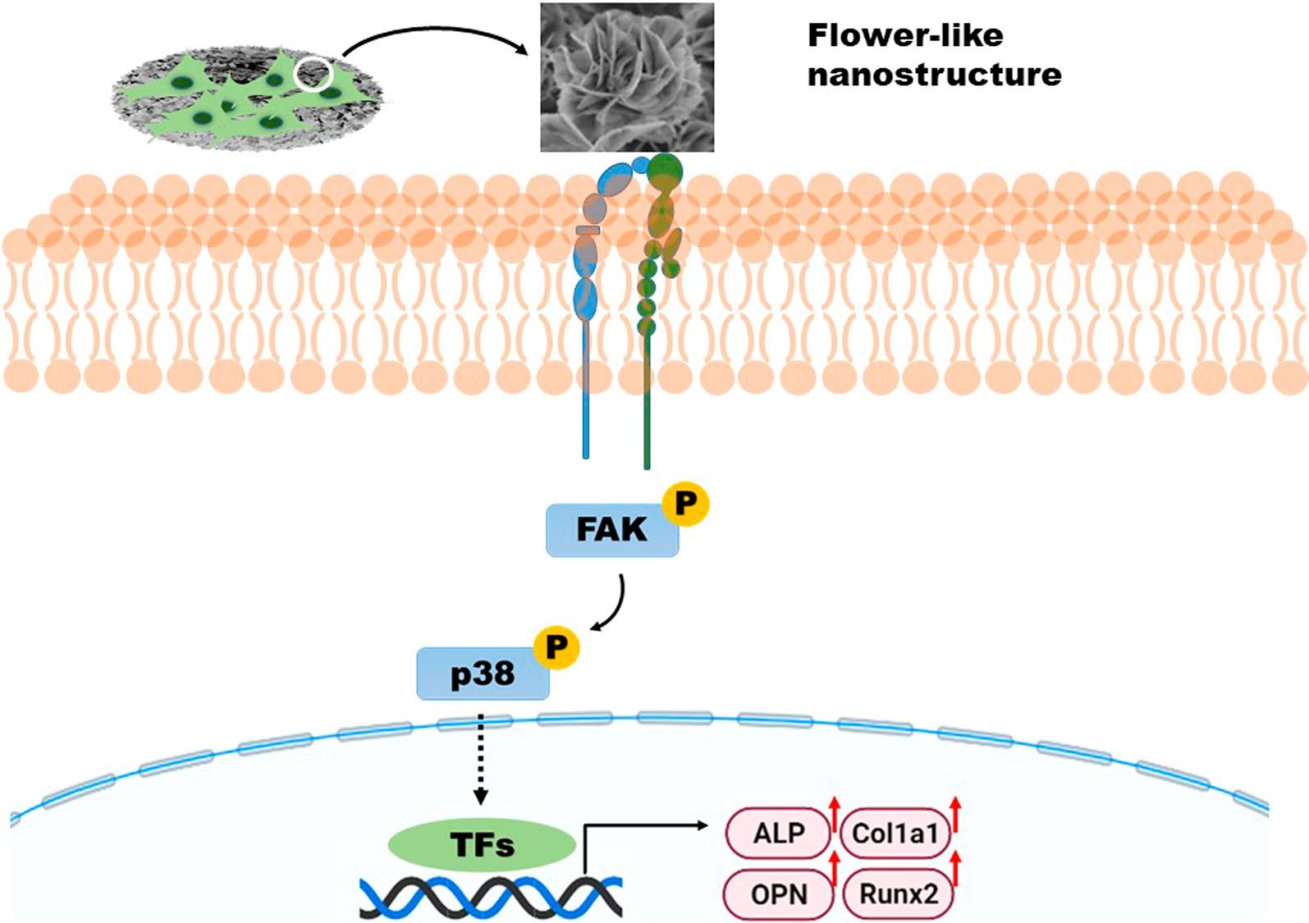
Schematic diagram of the effects of nCS bioceramics on osteogenic differentiation

Moreover, CS bioceramics could significantly promote osteogenic differentiation as an osteoimmunomodulatory agent to regulate macrophage polarization towards M2 and further enhance bone regeneration [56]. Besides, CS bioceramics could stimulate angiogenesis to further promote bone defects repair [57]. In the study, nCS bioceramics with flower-like nanostructures were successfully constructed and exhibited favorable protein loading capacity, suggesting the possibility that nCS bioceramics could be applied as a carrier to load chemical drugs for bone repair and treatment. In addition, flower-like nanostructure CS bioceramics could mimic the micro/nanostructures of natural bone, possess good degradability and exhibit the excellent ability of bone regeneration, providing a promising potential as an ideal bone implant substitutes in the clinical application (Fig. 10).

## Conclusion

Nanoscale structures mimic natural bone structure and promote the osteogenic differentiation and regeneration of new bone. In this study, flower-like nanostructures consisting of nanosheets were directly constructed in situ on CS bioceramics via hydrothermal treatment. In addition, the flower-like nanostructure significantly promoted cell behaviors, including adhesion, proliferation, and osteogenic differentiation. Compared with CS bioceramics with flat surfaces, CS bioceramics with flower-like nanostructures could accelerate new bone formation to promote bone repair and regeneration. Moreover, our results suggested that flower-like nanostructures could first induce cell adhesion and further activate the FAK/p38 signaling pathway to enhance osteogenic differentiation and bone regeneration. The present study indicated that flower-like nanostructures could be flexibly constructed directly on the surface of CS bioceramics and could accelerate the repair of bone defects, providing valuable inspiration for the design and preparation of bioactive materials for use in the fields of tissue regeneration and regenerative medicine.

## Supplementary Information

**Additional file 1.** Additional figures and tables.

## Data Availability

All data generated or analyzed during this study are included in this published article and its additional information files.

## Abbreviations

ALP: Alkaline phosphatase
BMP2: Bone morphogenetic protein 2
BMSC: Bone mesenchymal stem cells
BSA: Bovine serum albumin
Bsp: Bone sialoprotein
CLSM: Confocal laser scanning microscope
Col1a1: Collagen type I alpha 1
CS: Calcium silicate
EDS: Energy dispersive spectrometer
ERK: Extracellular signal-regulated kinase
FAK: Focal adhesion kinase
FESEM: Field emission scanning electron microscopy
FTIR: Fourier Transform Infrared Spectrometer
GAPDH: Glyceraldehyde-3-phosphate-dehydrogenase
H&E: Hematoxylin and eosin
HA: Hydroxyapatite
ICP-AES: Inductively coupled plasma atomic emission spectroscopy
JNK: Jun kinase
MAPK: Mitogen-activated protein kinase
nCS: Calcium silicate with flower-like nanostructure
Opn: Osteopontin
p-ERK: Phosphor-ERK
p-FAK: Phosphor-FAK
p-JNK: Phosphor-JNK
p-p38: Phosphor-p38
Runx2: Runt-related transcription factor-2
SD rat: Sprague–Dawley rat
SEM: Scanning electron microscopy
STEM: Scanning transmission electron microscope
TEM: Transmission electron microscope
TNF-α: Tumor necrosis factor α
XPS: X-ray photoelectron spectroscopy
XRD: X-ray diffraction analysis
β-TCP: β-Tricalcium phosphate
