# Supplementary material for: In situ construction of flower-like nanostructured calcium silicate bioceramics for enhancing bone regeneration mediated via FAK/p38 signaling pathway
Source: J Nanobiotechnology. 2022 Mar 27;20:162. doi: 10.1186/s12951-022-01361-5 (PMC8962168; doi:10.1186/s12951-022-01361-5)
Supplement: Supplementary file 1 — Additional file 1. Additional figures and tables. [file 12951_2022_1361_MOESM1_ESM.docx]

**In situ construction of flower-like nanostructured calcium silicate bioceramics for enhancing bone regeneration mediated via FAK/p38 signaling pathway**

Peng Mei^1^*, Shengjie Jiang^1^*, Lixia Mao^1^*, Yijia Zhou^2^, Kaijun Gu^1^, Chen Zhang^1,3^, Xudong Wang^1#^, Kaili Lin^1#^, Cancan Zhao^1#^, Min Zhu^1#^

^1^ Department of Oral & Cranio-maxillofacial Surgery, Shanghai Ninth People’s Hospital, College of Stomatology, Shanghai Jiao Tong University School of Medicine, National Clinical Research Center for Oral Diseases, Shanghai Key Laboratory of Stomatology & Shanghai Research Institute of Stomatology, Shanghai, China.

^2^ Department of General Dentistry, Shanghai Ninth People’s Hospital, College of Stomatology, Shanghai Jiao Tong University School of Medicine, National Clinical Research Center for Oral Diseases, Shanghai Key Laboratory of Stomatology & Shanghai Research Institute of Stomatology, Shanghai, China.

^3^ Shanxi Medical University School and Hospital of Stomatology, Taiyuan, China

* Co-first author.

^#^Corresponding authors.

E-mail: xudongwang70@hotmail.com (Xudong Wang),

lklecnu@aliyun.com (Kaili Lin),

cczhaozita@126.com (Cancan Zhao),

zminnie@126.com (Min Zhu).

**Table S1 Primers used for the target genes**

| Gene | Primer (F = forward; R = reverse) |
| --- | --- |
| BSP | F: 5′AGAAAGAGCAGCACGGTTGAGT3′  R: 5′ GACCCTCGTAGCCTTCATAGCC3′ |
| Runx-2 | F:5’CACAAGTGCGGTGCAAACTT3’  R:5’AAGAGGCTGTTTGACGCCAT3’ |
| OPN | F:5’AGACTGGCAGTGGTTTGCTT3’  R:5’AGTGTTTGCTGTAATGCGCC3’ |
| Col1a1 | F:5’GGAGAGAGCATGACCGATGG3’  R:5’GGGACTTCTTGAGGTTGCCA3’ |
| GAPDH | F:5’GGCACAGTCAAGGCTGAGAATG3’  R:5’ATGGTGGTGAAGACGCCAGTA3’ |

Table S2: Element ratio of CS and nCS.

| CS | Atomic % | nCS | Atomic % |
| --- | --- | --- | --- |
| C 1s | 23.76 | C 1s | 27.07 |
| Ca 2p | 12.26 | Ca 2p | 11.22 |
| O 1s | 48.11 | O 1s | 45.2 |
| Si 2p | 15.87 | Si 2p | 16.51 |


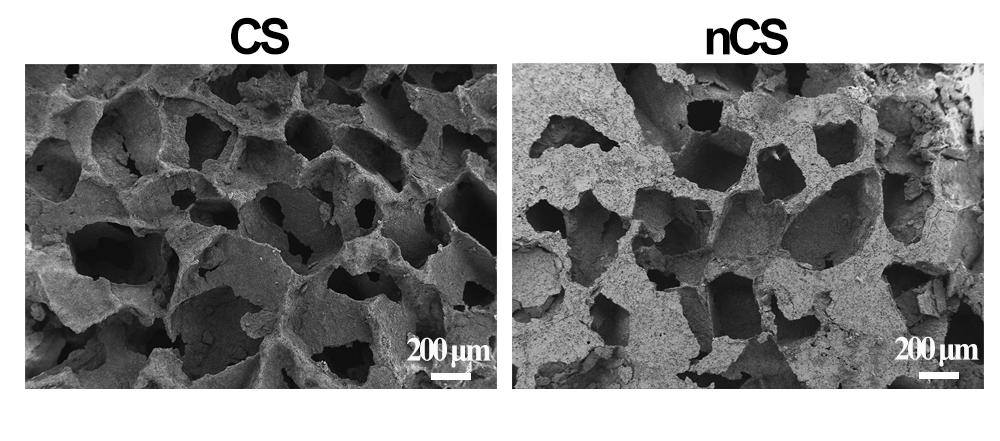


Fig S1 FESEM images of the CS and nCS bioceramic scaffolds. (Scale bar=200 μm)


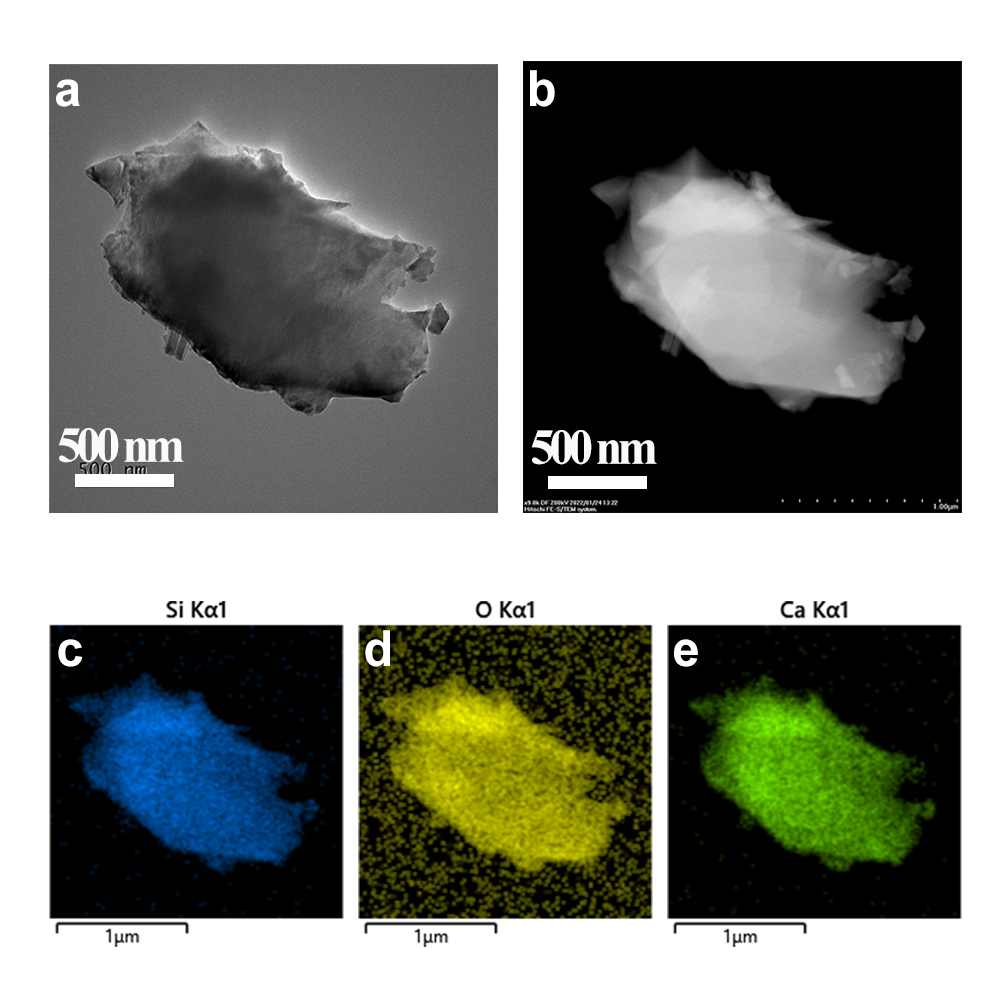


Fig. S2 (a-b) TEM and STEM images of flower-like nanostructures on nCS bioceramics. (c-e) STEM-EDS mapping of flower-like nanostructures on nCS bioceramics.


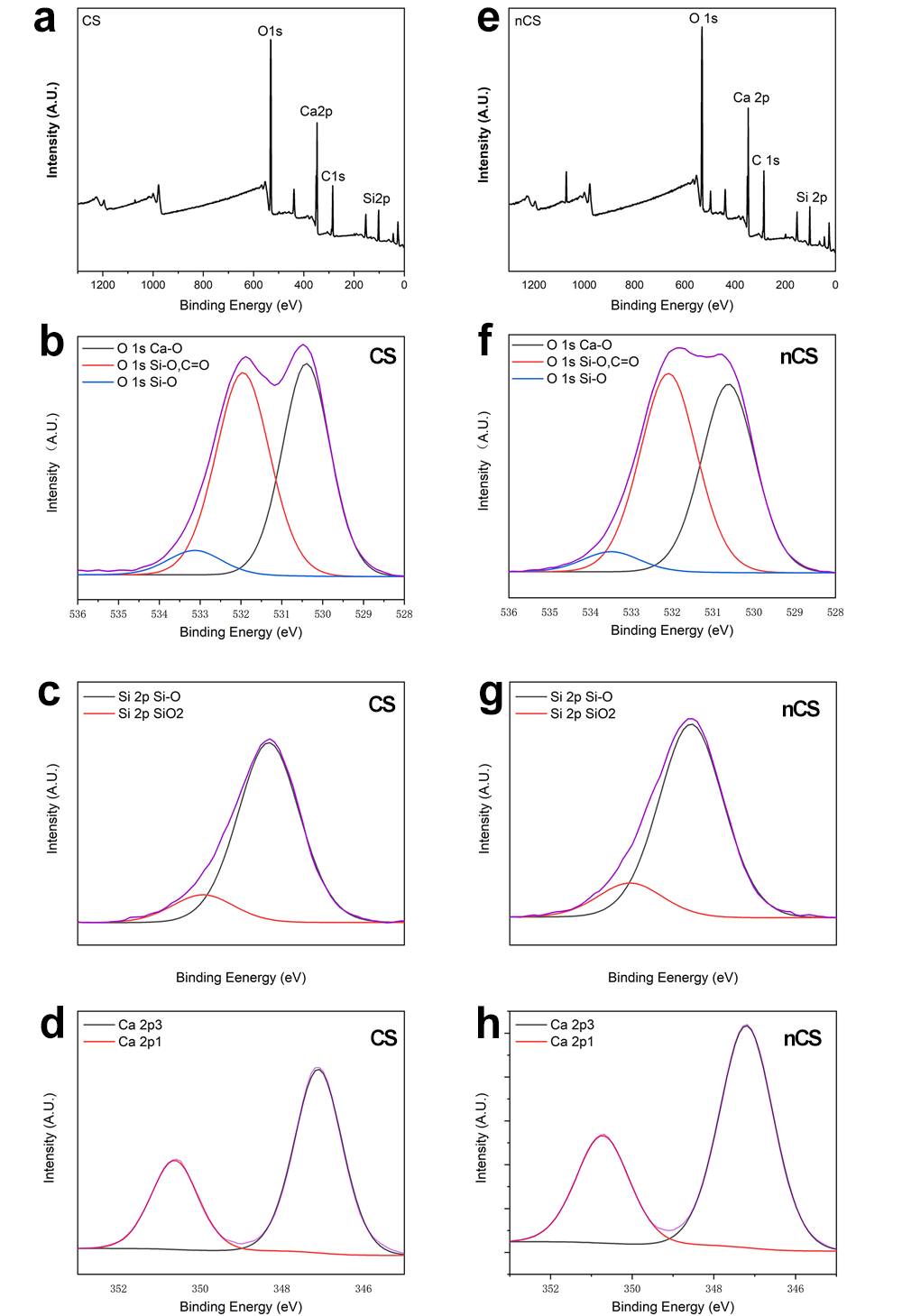


Fig. S3 (a) XPS survey scan and (b) O 1s, (c) Si 2p, (d) Ca 2p3 and Ca 2p1 XPS spectra of CS. (e) XPS survey scan and (f) O 1s, (g) Si 2p, (h) Ca 2p3 and Ca 2p1 XPS spectra of nCS.


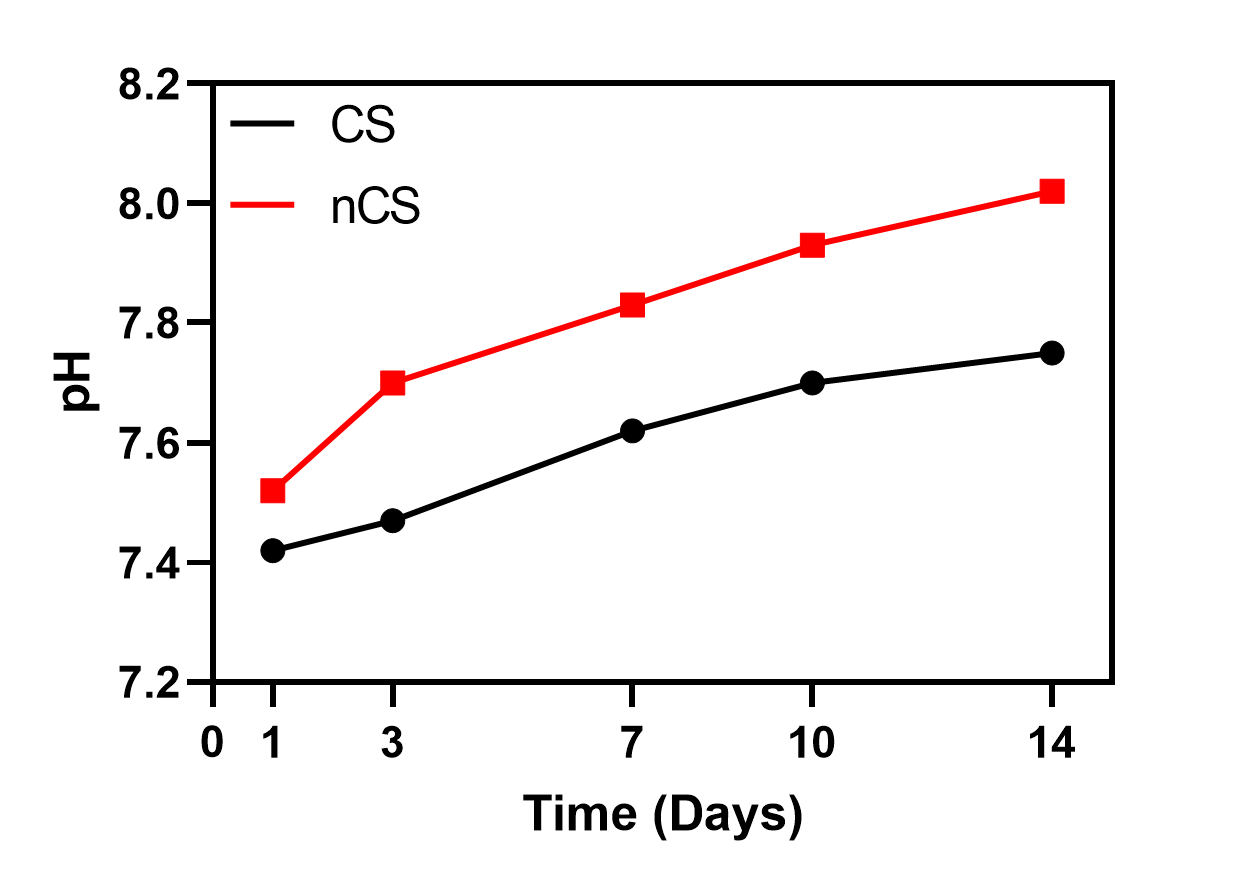


Fig. S4 The pH value of Tris-HCl in which the CS and nCS soaked for 14 days.


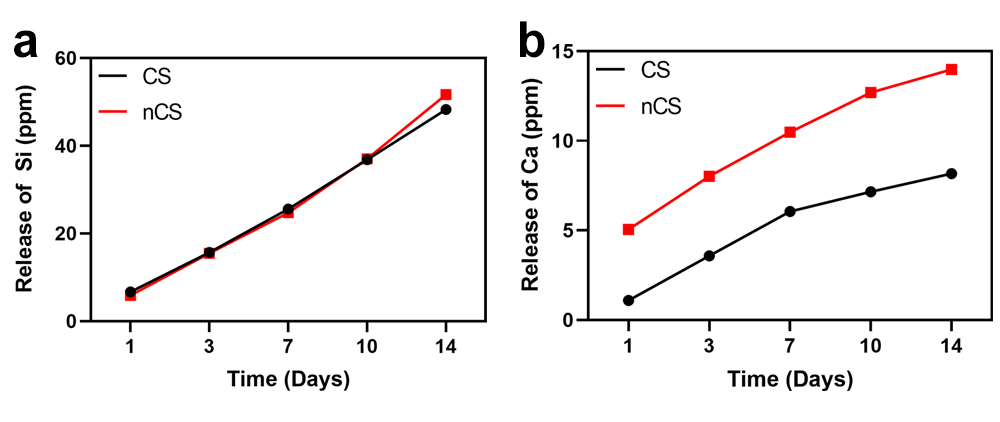


Fig. S5 The release curve of Si (a) and Ca (b) ions from CS and nCS bioceramics in PBS for 14 days.


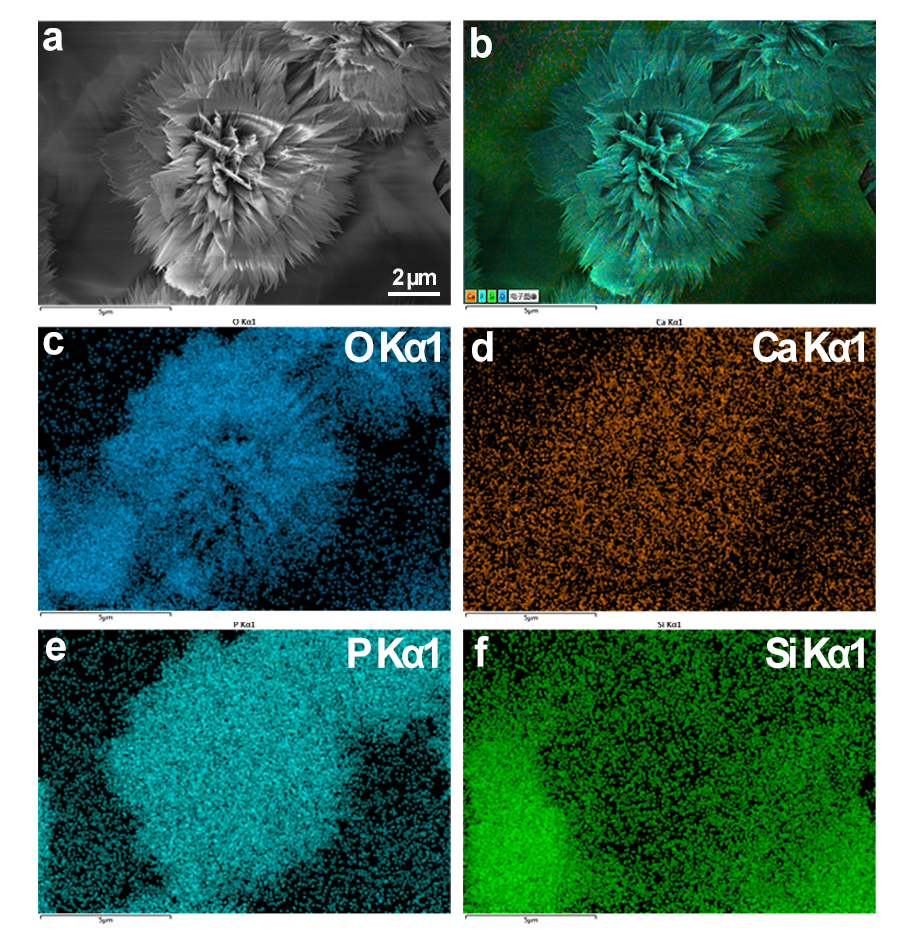


Fig. S6 CS samples soaked in PBS solution for 14 days. (a) SEM image of CS. (b-f) mapping of CS. Scale bar=2 μm


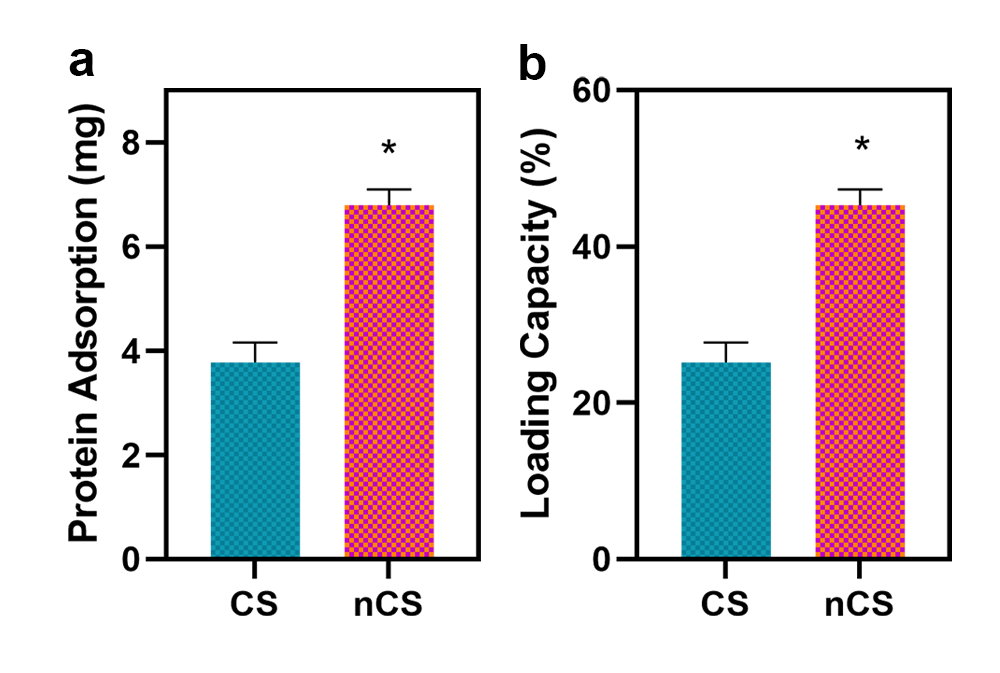


Fig. S7 (a) The amount of BSA adsorbed and (b) the BSA loading capacity on CS and nCS bioceramics. (*p < 0.05)


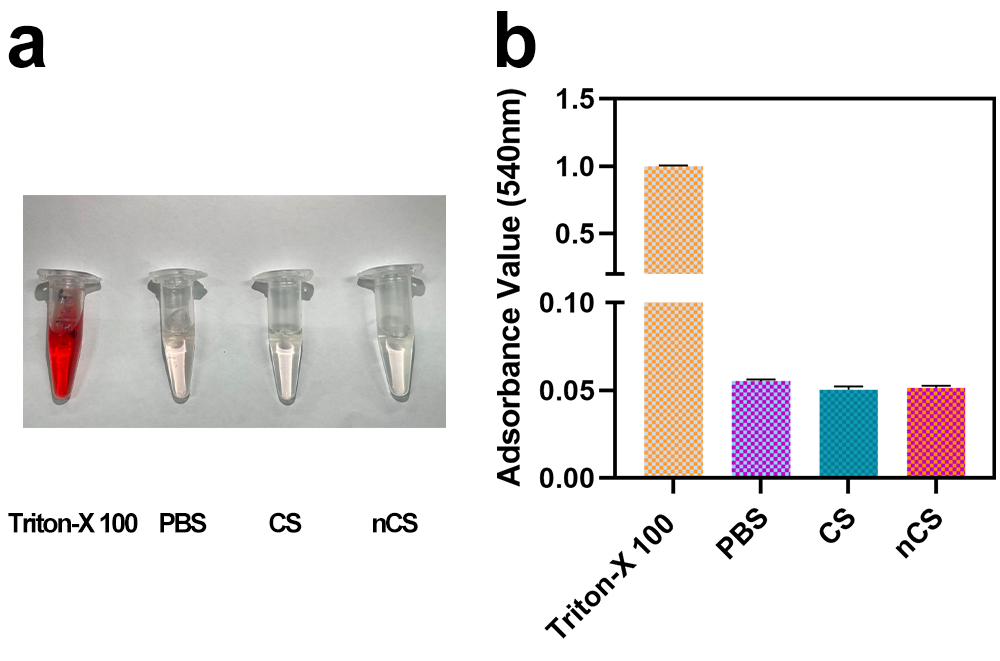


Fig. S8 (a) Photograph of blood cells incubated with Triton-X 100, PBS, CS and nCS for 1 h. (b) Quantitative analysis of hemolytic test.


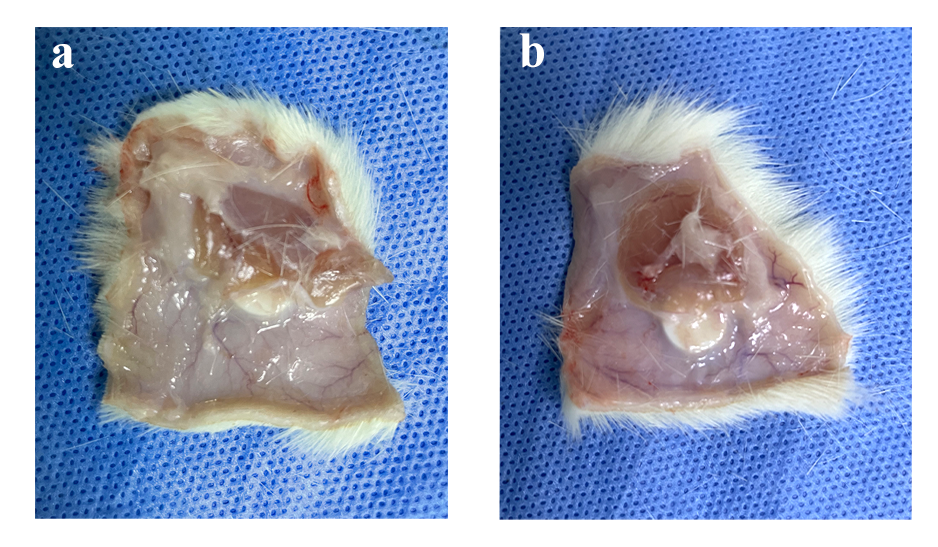


Fig. S9 Digital photographs of (a) CS and (b) nCS implanted into subcutaneous for 2 weeks.


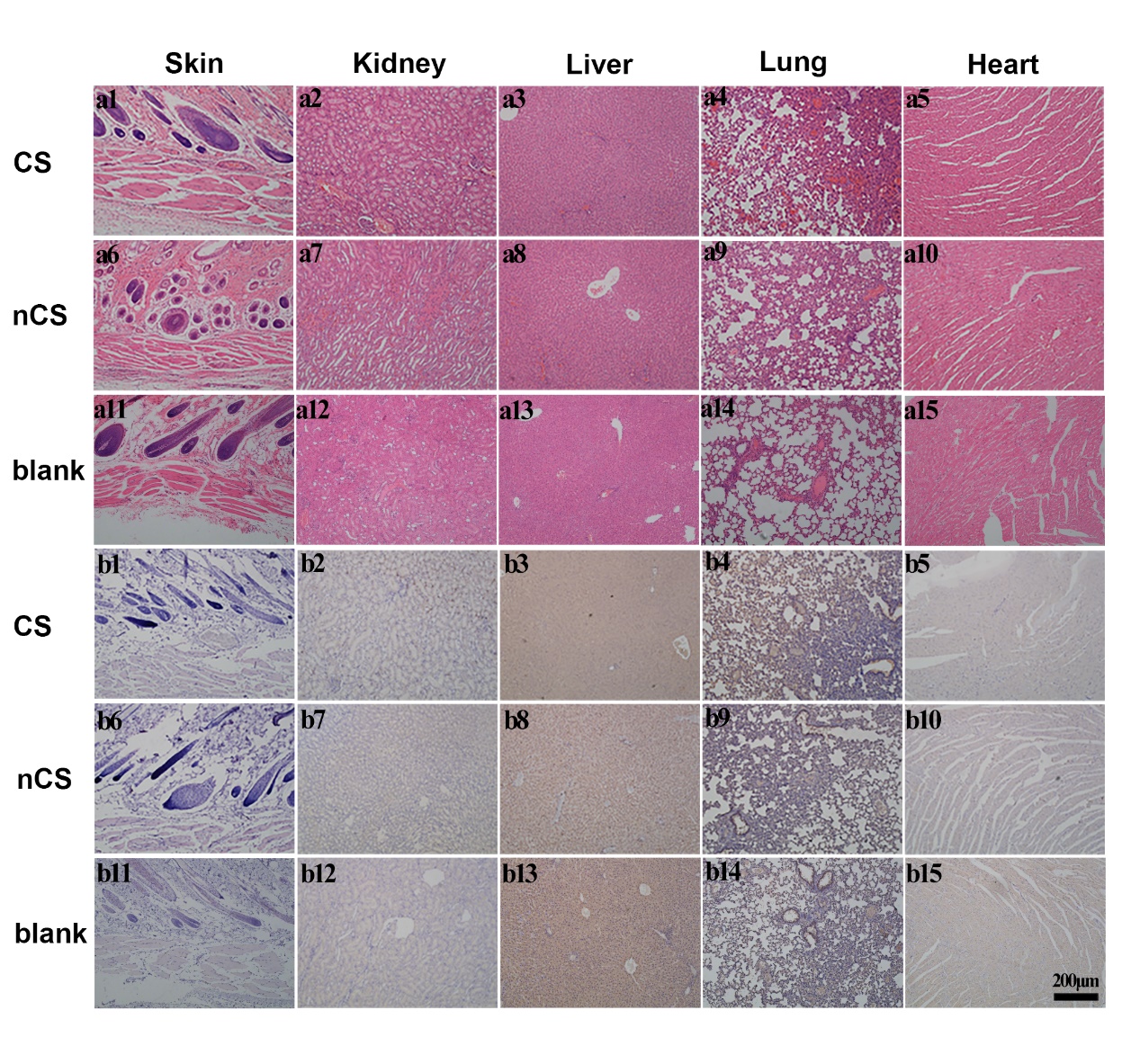


Fig. S10 (a1-a15) H&E staining and (b1-b15) immunohistochemical staining of TNF-α of skin which was removed bioceramics and important organs (Heart, Kidney, Liver, and Lung) in CS and nCS groups after being implanted into subcutaneous for 2 weeks. Scale bar=200 μm
